# Polydopamine Nanoparticles as an Organic and Biodegradable Multitasking Tool for Neuroprotection and Remote Neuronal Stimulation

**DOI:** 10.1021/acsami.0c05497

**Published:** 2020-07-22

**Authors:** Matteo Battaglini, Attilio Marino, Alessio Carmignani, Christos Tapeinos, Valentina Cauda, Andrea Ancona, Nadia Garino, Veronica Vighetto, Gabriele La Rosa, Edoardo Sinibaldi, Gianni Ciofani

**Affiliations:** † Smart Bio-Interfaces, Istituto Italiano di Tecnologia, Viale Rinaldo Piaggio 34, 56025 Pontedera, Italy; ‡ The Biorobotics Institute, Scuola Superiore Sant’Anna, Viale Rinaldo Piaggio 34, 56025 Pontedera, Italy; § Department of Applied Science and Technology, Politecnico di Torino, Corso Duca Degli Abruzzi 24, 10129 Torino, Italy; ∥ Nanochemistry, Istituto Italiano di Tecnologia, Via Morego 30, 16163 Genova, Italy; ⊥ Bioinspired Soft Robotics, Istituto Italiano di Tecnologia, Viale Rinaldo Piaggio 34, 56025 Pontedera, Italy

**Keywords:** neurodegenerative diseases, polydopamine, antioxidant nanoparticles, near-infrared stimulation, neuronal stimulation

## Abstract

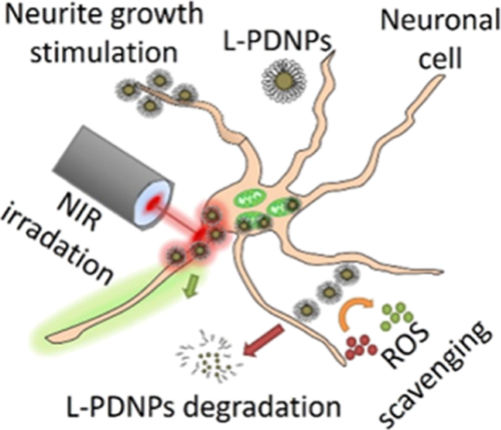

Oxidative stress represents a common issue in most neurological diseases, causing severe impairments of neuronal cell physiological activity that ultimately lead to neuron loss of function and cellular death. In this work, lipid-coated polydopamine nanoparticles (L-PDNPs) are proposed both as antioxidant and neuroprotective agents, and as a photothermal conversion platform able to stimulate neuronal activity. L-PDNPs showed the ability to counteract reactive oxygen species (ROS) accumulation in differentiated SH-SY5Y, prevented mitochondrial ROS-induced dysfunctions and stimulated neurite outgrowth. Moreover, for the first time in the literature, the photothermal conversion capacity of L-PDNPs was used to increase the intracellular temperature of neuron-like cells through near-infrared (NIR) laser stimulation, and this phenomenon was thoroughly investigated using a fluorescent temperature-sensitive dye and modeled from a mathematical point of view. It was also demonstrated that the increment in temperature caused by the NIR stimulation of L-PDNPs was able to produce a Ca^2+^ influx in differentiated SH-SY5Y, being, to the best of our knowledge, the first example of organic nanostructures used in such an approach. This work could pave the way to new and exciting applications of polydopamine-based and of other NIR-responsive antioxidant nanomaterials in neuronal research.

## Introduction

Reactive oxygen species (ROS) are one of the main protagonists in neurological diseases, being responsible or at least involved in many of the cellular damages which are typical of brain dysfunctions.^1^ The relation between ROS and neurological disorders is extraordinarily complicated and still poorly understood; however, the literature on the topic makes it evident that ROS play a significant role in many of the most common brain diseases, including Alzheimer’s disease, Parkinson’s disease, ischemic stroke, multiple sclerosis, and Huntington’s disease.^1−6^ ROS are necessary for the homeostasis of physiological functions, being involved in mitochondrial respiration, autophagy, energy production, and other regulatory pathways;^7^ however, an overproduction of ROS not counterbalanced by endogenous antioxidant mechanisms may lead to the harmful condition of oxidative stress.

Neurons are one of the primary victims of oxidative stress-induced damages because of the high metabolic level of the brain that generates a high level of ROS, and because of the relatively low level of antioxidant protection mechanisms present at the neuronal level.^3,8^ High levels of ROS can affect several physiological cellular functions, being able to damage proteins, cell membranes, and mitochondria.^1,7^ Mitochondria, in particular, play a central role in the ROS-induced damage because of cellular respiration; furthermore, they are the main ROS generators in the cells, besides being one of the main targets of ROS damage.^9^ Oxidative stress and ROS-induced cellular dysfunctions are also interconnected in a self-renewing cycle, in which the damages induced by ROS can provoke the production of even more ROS, which in turn can further exacerbate the already compromised cellular components.^1^ The endpoint of this cycle is commonly the loss of neuronal cell functions and the subsequent cell death.

Antioxidant nanomaterials have been studied as a countermeasure to oxidative stress conditions in neurological diseases; they can be classified into two main categories, namely, drug delivery systems loaded with antioxidant moieties and nanozymes with intrinsic antioxidant properties.^1^ Usually, drug delivery systems loaded with antioxidant molecules present the same drawbacks of the loaded cargo, generally being only able to scavenge single specific species of ROS and having low antioxidant capabilities compared to inorganic nanozymes. On the other hand, inorganic nanoparticles like cerium oxide nanoparticles (nanoceria), platinum-based nanomaterials, or manganese oxide nanoparticles present higher antioxidant capacities and are active on a broad spectrum of different ROS.^10−13^ Our group, in particular, broadly investigated the use of nanoceria in biomedical applications because of their unique antioxidant capabilities, including applications in neuronal cells, mitochondrial protection, muscle cells exposed to microgravity, and obesity.^14−20^ However, one of the significant drawbacks that strongly limits the possibility to exploit nanoceria and other nonorganic antioxidants in human clinical applications is their inorganic nature that hinders their degradability and clearance in living organisms. As an example, previous works have shown how traces of nanoceria could be found in animals even 90 days after the nanoparticle injection.^21^ Polydopamine nanoparticles (PDNPs) could represent an optimal compromise between the two classes of nano-antioxidant materials, being a fully organic, biodegradable, and biocompatible class of nanostructures with exceptional antioxidant abilities.^22^ Antioxidant capabilities of polydopamine-based materials have provided promising results in the treatment of inflammation, Parkinson’s disease, and periodontal diseases.^23−26^

In this work, PDNPs with a lipid coating (L-PDNPs) have been investigated as a multitasking platform able to counteract ROS-induced damages in neuron-like cells. After a detailed material characterization in terms of morphology, size, *in vitro* antioxidant properties, and porosity, the ability of L-PDNPs to prevent ROS accumulation was analyzed on SH-SY5Y subjected to pro-oxidative stimulation (induced by *tert*-butyl hydroperoxide, TBH). Mitochondrial protective effects of L-PDNPs were assessed in terms of prevention of ROS-induced mitochondrial morphology modification and membrane potential (ΔΨ_m_) loss. The ability of L-PDNPs to stimulate neurite outgrowth was also measured and quantified together with the effect of different pH and ROS concentrations on L-PDNP degradation.

Another interesting feature of PDNPs, and in general of polydopamine-based materials, is their ability to convert near-infrared (NIR) radiation in thermal energy generating heating. This ability has been investigated in relatively recent times, with a few applications mainly regarding the use of PDNPs in photothermal ablation of cancer cells.^27,28^ In this work, the photothermal conversion ability of PDNPs was investigated as a tool to control cellular function in a nondisruptive approach, tuning the temperature and the activation of neuronal cells. This strategy represents, to the best of our knowledge, the very first example where a fully organic nanomaterial has been used in NIR-mediated heating and stimulation of neuronal cells. The ability of L-PDNPs to increase intracellular temperature upon NIR irradiation was investigated using a fluorescent temperature-sensitive dye, also providing a tool to quantify the NIR-mediated heating effect at the cellular level. A theoretical model of temperature increment because of L-PDNP photothermal conversion was developed, which was calibrated thanks to the data provided by the temperature-sensitive dye. Lastly, the ability of L-PDNPs to stimulate neuronal activity was assessed through calcium imaging, measuring the variations of Ca^2+^ content in cells upon L-PDNP-mediated NIR thermal stimulation.

Collected results suggest a high potential for PDNPs in neuroscience and neurological disease treatment, combining into a single biocompatible and biodegradable nanostructure a high antioxidant and neuroprotective action, an intrinsic ability to stimulate neurite outgrowth, and a platform for the NIR-mediated fine tuning of cellular temperature and activity.

## Results and Discussion

### Nanoparticle Characterization

**Figure 1:**
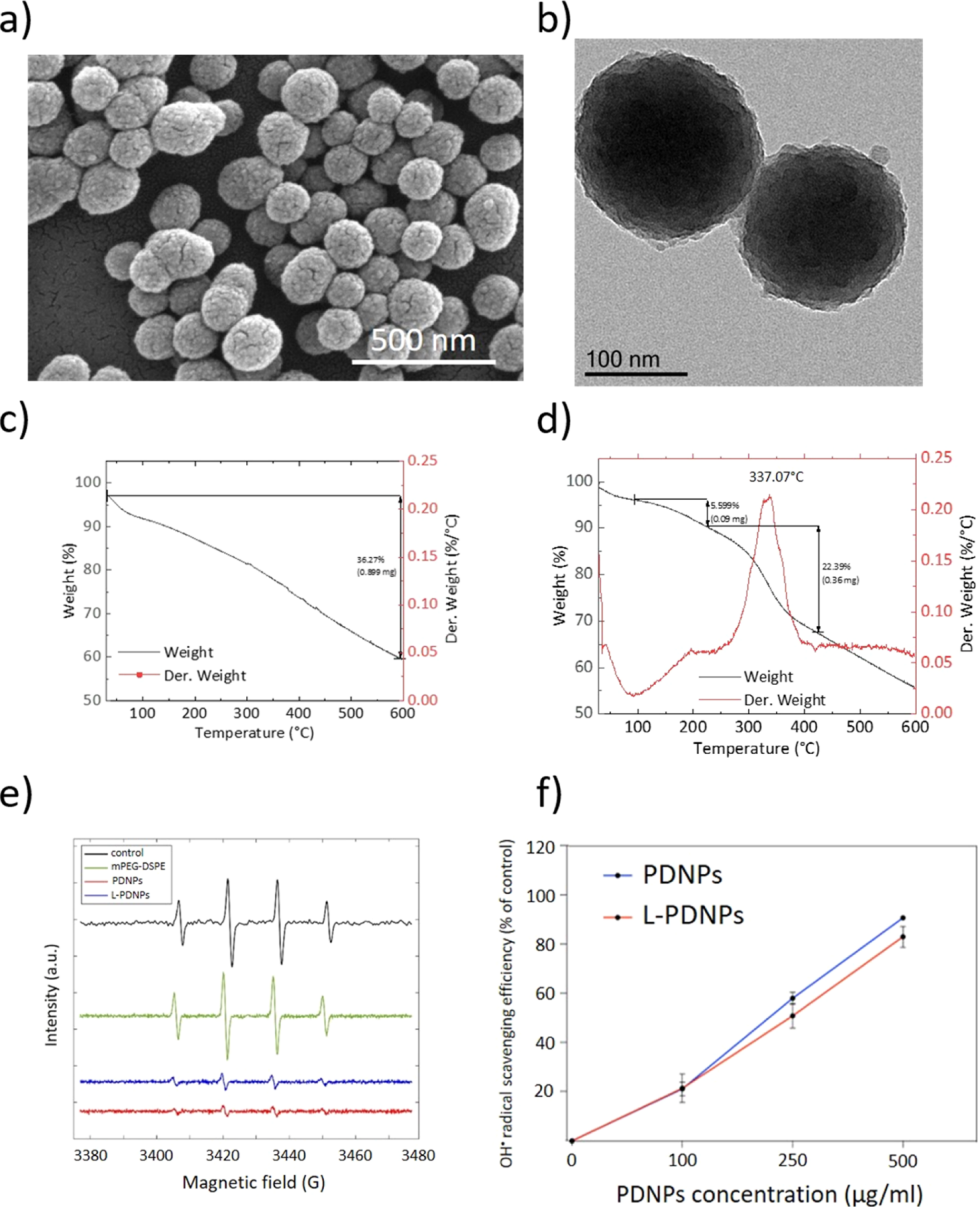
Characterization of L-PDNPs. Representative (a) SEM and (b) TEM images of L-PDNPs showing spherical morphology and uniform size. In (c) and (d) TGA of PDNPs and L-PDNPs is reported; derivative plots are shown in red. (e) Representative EPR spectra of mPEG-DSPE, PDNPs, and L-PDNPs (blue, red, and green trace, respectively). (f) OH^•^ scavenging efficiency obtained for L-PDNPs (blue trace) and PDNPs (red trace) samples expressed as percentage of the radicals in the absence of nanoparticles.

L-PDNPs were prepared through a Stöber process. Morphological analysis was carried out both by scanning electron microscopy (SEM) and transmission electron microscopy (TEM) imaging (Figure 1a,b, respectively). Both techniques showed the presence of spherical structures with uniform size and morphology and an average diameter of 170 ± 30 nm. These results are similar to what already described in the literature by other works using an analogous synthesis method.^23^

The coating with 1,2-distearoyl-*sn*-glycerol-3-phosphoethanolamine conjugated with methoxyl poly(ethylene glycol) (mPEG-DSPE) was quantified through thermogravimetric analysis (TGA) comparing the degradation profile obtained for PDNPs, L-PDNPs, and mPEG-DSPE (Figures 1c,d and S1 respectively). Despite the fact that both mPEG-DSPE and PDNPs are organic and thus the heating-mediated degradative processes are more complex compared to mixtures of organic/inorganic materials, we were able to provide an estimate of the mPEG-DSPE content (mPEG-DSPE ≅ 5% of the total particles weight). When TGA analysis was performed on plain PDNPs, 60% of the initial mass was lost after the degradative process from 30 to 600 °C. L-PDNPs, on the other hand, presented a 55% of residual mass after the same heating profile. Because, as shown in Figure S1, mPEG-DSPE was completely degraded after the heating from 30 to 600 °C, we deduced that the 5% difference observed between PDNP and L-PDNP samples can be attributed to the lipid component.

To evaluate the antioxidant activity of L-PDNPs, hydroxyl radicals were generated *in situ* by Fenton reaction (Fe^2+^ + H_2_O_2_ → Fe^3+^ + OH^•^ + HO^–^). This was chosen because of its high OH radical generation efficiency and reproducibility.^29^ Because of a very short half-life of hydroxyl radicals, the spin-trap technique coupled with electron paramagnetic resonance (EPR) spectroscopy was used. The oxidation of the spin trap 5,5-dimethyl-1-pyrroline-*N*-oxide (DMPO) by hydroxyl radicals was studied, and results are shown in Figure 1e,f. After 5 min from the start of the Fenton reaction, the EPR spectrum (black curve), as shown in Figure 1e, was obtained. This shows the typical four peaks corresponding to the spin-adduct DMPO-OH, resulting from the reaction of the hydroxyl radicals with the spin-trap DMPO. The addition of PDNPs at a concentration of 500 μg/mL protected the spin-trap DMPO from oxidation by the hydroxyl radicals, eventually resulting in significantly lower peaks in the DMPO-OH spectrum (Figure 1e, red curve). To understand whether PDNPs could preserve their OH radical scavenging ability once coated with a lipid shell, L-PDNPs at the same concentration were analyzed. As shown in Figure 1e (blue curve), L-PDNPs effectively protected DMPO from oxidation as efficiently as PDNPs, thus showing that the lipid shell does not modify the antioxidant properties of PDNPs. Free mPEG-DSPE, indeed, did not contribute to the antioxidant property of the construct, as shown in Figure 1e, green curve. To further investigate whether the antioxidant property was concentration-dependent, different concentrations of PDNPs and L-PDNPs were tested. As shown in Figure 1f, by increasing the concentration of nanoparticles up to 500 μg/mL, the number of hydroxyl radicals trapped increased, reaching an OH^•^ radical scavenging efficiency of 90% for PDNPs and 85% for L-PDNPs. Together, these results show that PDNPs have strong antioxidant properties, not affected by the lipid functionalization.

L-PDNPs were also analyzed through dynamic light scattering (DLS) measurement and showed an average hydrodynamic diameter of 213.2 ± 6.9 nm (Figure S2a) with a polydispersity index (PDI) of 0.04 ± 0.02 and an average surface *Z*-potential of −43.1 ± 0.4 mV (Figure S2b). Moreover, the porosity of L-PDNPs was analyzed using the Brunauer–Emmett–Teller (BET) method (Figure S2c,d), showing a surface area of 29.89 m^2^/g, pore volume of 0.274 cm^3^/g, and pore maximum diameter of 3.83 nm.

### Degradation of L-PDNPs

**Figure 2:**
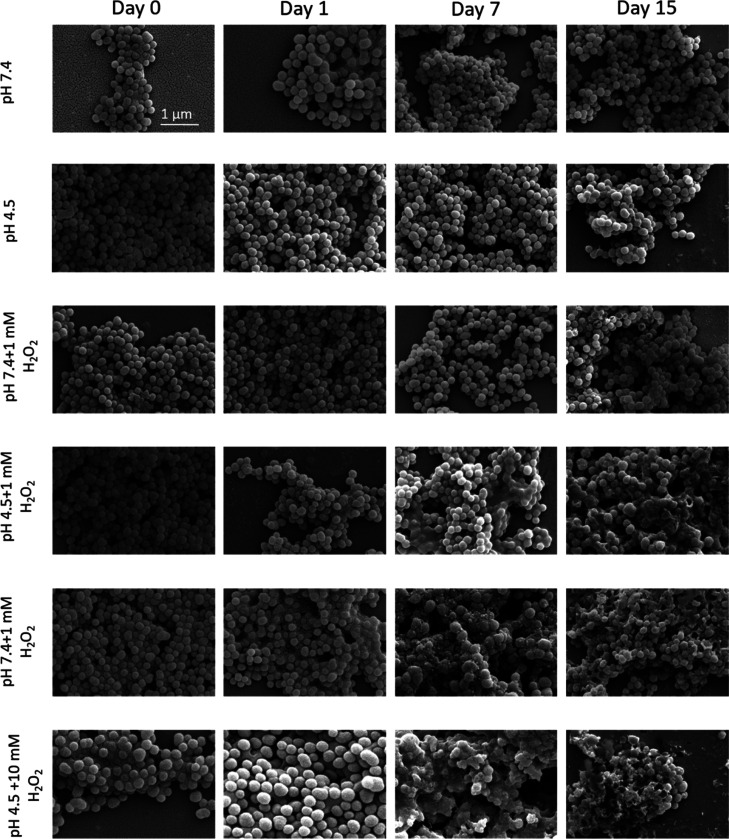
Representative SEM images showing the degradation of L-PDNPs at different pH values (7.4 and 4.5) and in the presence of H_2_O_2_ at various concentrations (0, 1, and 10 mM).

The degradation profile of L-PDNPs was qualitatively studied with SEM observation at two different pH values (pH 7.4 and 4.5) and with or without the presence of H_2_O_2_ (1 and 10 mM), aiming at mimicking *in vivo* conditions of oxidative stress at the level of the diseased tissue. The results presented in Figure 2 show no degradation even after 15 days at pH 7.4 (physiological pH) and pH 4.5 (typical of the acidic organelles). On the other hand, the addition of 1 mM of H_2_O_2_ leads to an observable degradation at day 15. A higher and faster degradation can be observed when the concentration of H_2_O_2_ is increased to 10 mM for both pH values: the first signs of degradation start at day 7 and become more evident at day 15. This study shows that the degradation of L-PDNPs is mostly related to the presence of H_2_O_2_ and is not qualitatively affected by different pH values. This can be attributed to the scavenging ability of L-PDNPs that interacts with ROS like H_2_O_2_ and OH^•^, leading ultimately to their degradation. Although SEM is not a quantitative technique, it can still provide sounding data about the degradation profile.

### Nanoparticle–Cell Interactions

L-PDNP effects on cell viability were assessed on differentiated SH-SY5Y through PicoGreen assay. Fluorescence levels of cells treated with different concentrations of L-PDNPs (0, 31.25, 62.5, 125, and 250 μg/mL) did not present any statistically significant difference (*p* > 0.05) with respect to the corresponding control at the same time points (24 or 72 h, red and green columns of Figure S3a, respectively), suggesting good biocompatibility of the nanoparticles in the tested concentration range. This conclusion was further confirmed through a LIVE/DEAD assay; in particular, no statistically significant difference (*p* > 0.05) was observed in terms of dead cell number among all tested conditions. Quantitative data of LIVE/DEAD assay are reported in Figure S3b, while representative fluorescence images are depicted in Figure S4.

**Figure 3:**
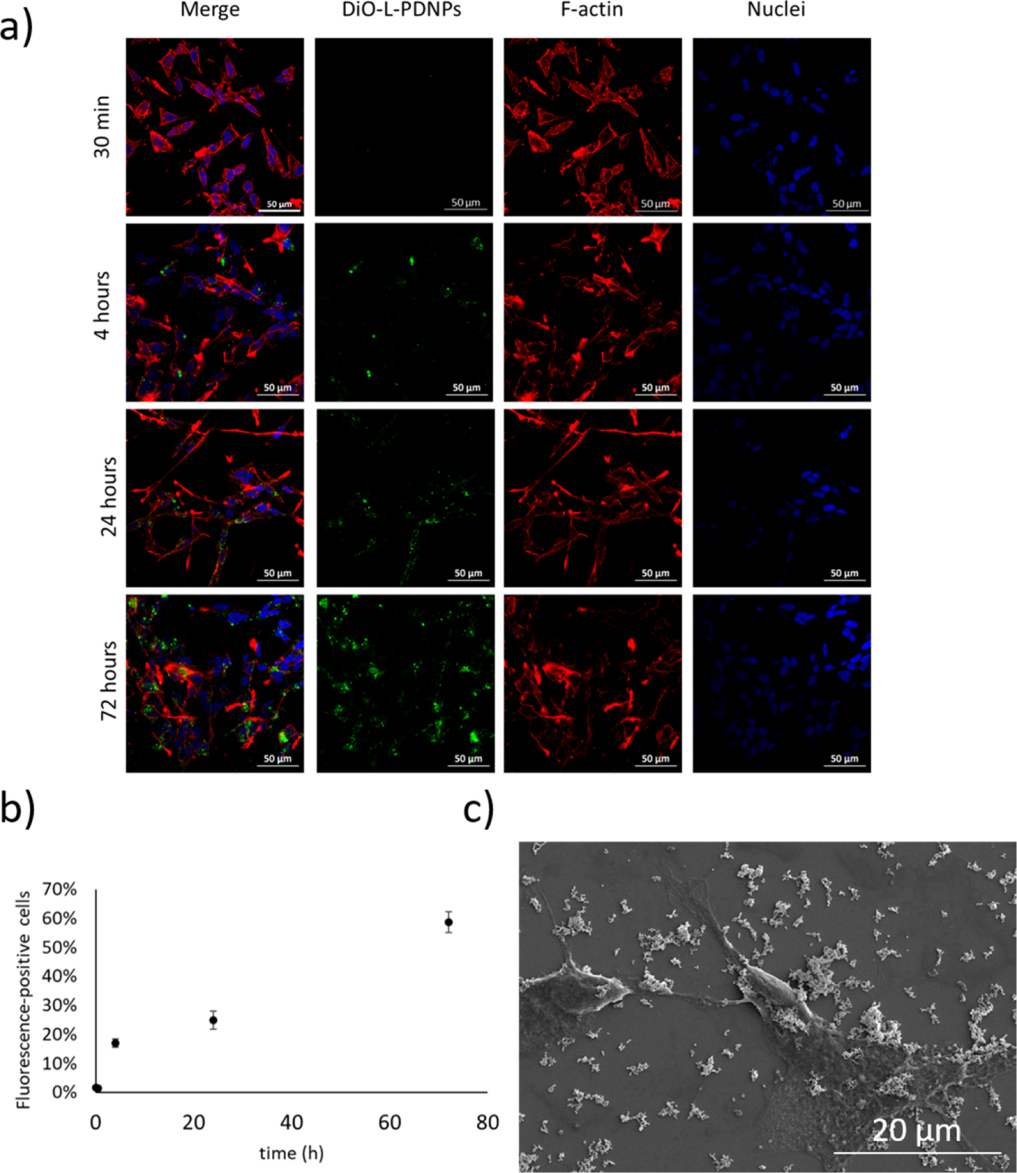
Analysis of L-PDNP interaction with differentiated SH-SY5Y cells. (a) Internalization of DiO-stained L-PDNPs at different time points (0.5, 4, 24, and 72 h; DiO-L-PDNPs in green, F-actin in red, nuclei in blue). (b) Flow cytometry analysis of differentiated SH-SY5Y incubated with DiO-L-PDNPs at the same time points (*n* = 3). (c) Representative SEM image showing L-PDNPs associated with the cellular surface of SH-SY5Y after 72 h of incubation.

Confocal microscopy analysis showed a time-dependent internalization of fluorescently-labeled DiO-L-PDNPs by differentiated SH-SY5Y after 4, 24, and 72 h of incubation, while no significant internalization was present after 30 min of treatment (2D images shown in Figure 3a, 3D rendering shown in Figure S5). Flow cytometry analysis of cells incubated with DiO-L-PDNPs confirmed these data (Figures 3b and S6), with the relative fluorescence level of cells treated for 30 min being not statistically different (*p* > 0.05) from control cells; instead, cultures treated for 4, 24, and 72 h showed cell populations shifted toward statistically significant (*p* < 0.001) higher fluorescence values (Figure 3b). In particular, we found 16.69 ± 1.42% of fluorescence-positive cells after 4 h of treatment with DiO-L-PDNPs, 24.83 ± 3.09% after 24 h, and 58.60 ± 3.60% after 72 h. SEM observation of cells treated for 72 h with L-PDNPs showed also the presence of particles associated with the external surface of the cell membrane (Figure 3c).

The intracellular fate of the nanoparticles was also investigated analyzing the co-localization of DiO-L-PDNPs with lysosomes and mitochondria (Figures S7 and S8, respectively). Most DiO-L-PDNPs showed to be uptaken by lysosomes (Figure S7a), with the Pearson correlation coefficient between particle and lysosome fluorescence channels being equal to 0.554 ± 0.057 after 4 h, 0.622 ± 0.042 after 24 h, and 0.682 ± 0.018 after 72 h (Figure S7b). Conversely, no significant co-localization with mitochondria was found (Figure S8).

All combined, these analyses showed that L-PDNPs are both internalized by cells and associated to the external cell surface, and both subpopulations could contribute to the effects described in the following.

The ability of L-PDNPs to be internalized by brain endothelial cells and to cross a blood–brain barrier (BBB) model was preliminary assessed with a transwell system *in vitro*. The model, based on a monolayer of bEnd.3 cells, was characterized in terms of the ZO-1 expression by immunostaining and, as shown in Figure S9a, cells seeded on porous membranes qualitatively showed high expression of ZO-1, suggesting the formation of tight junctions. The BBB *in vitro* model was further characterized by measuring its trans-endothelial electrical resistance (TEER; 70 Ω cm^2^) and by assessing the passage of a fluorescent tracer (FITC-dextran 70 kDa). In particular, as shown in Figure S9b, the presence of the bEnd.3 cells hindered the ability of FITC-dextran to reach the basolateral (bottom) side of the transwell, suggesting the formation of a tight cell monolayer. DiO-L-PDNPs showed the ability to be internalized by bEnd.3 cells, as shown in Figure S10. Furthermore, we estimated through absorbance measurements that the amount of L-PDNPs able to cross the bEnd.3 cells layer and to reach the basolateral side of the transwell was 25.0 ± 1.7% (w/w) of the total amount added to the apical side of the insert. This preliminary result suggests the ability of L-PDNPs to cross a simple BBB *in vitro* model, most probably owing to their lipid coating.^19,30−32^

### Antioxidant Effects on Neuronal Cells

**Figure 4:**
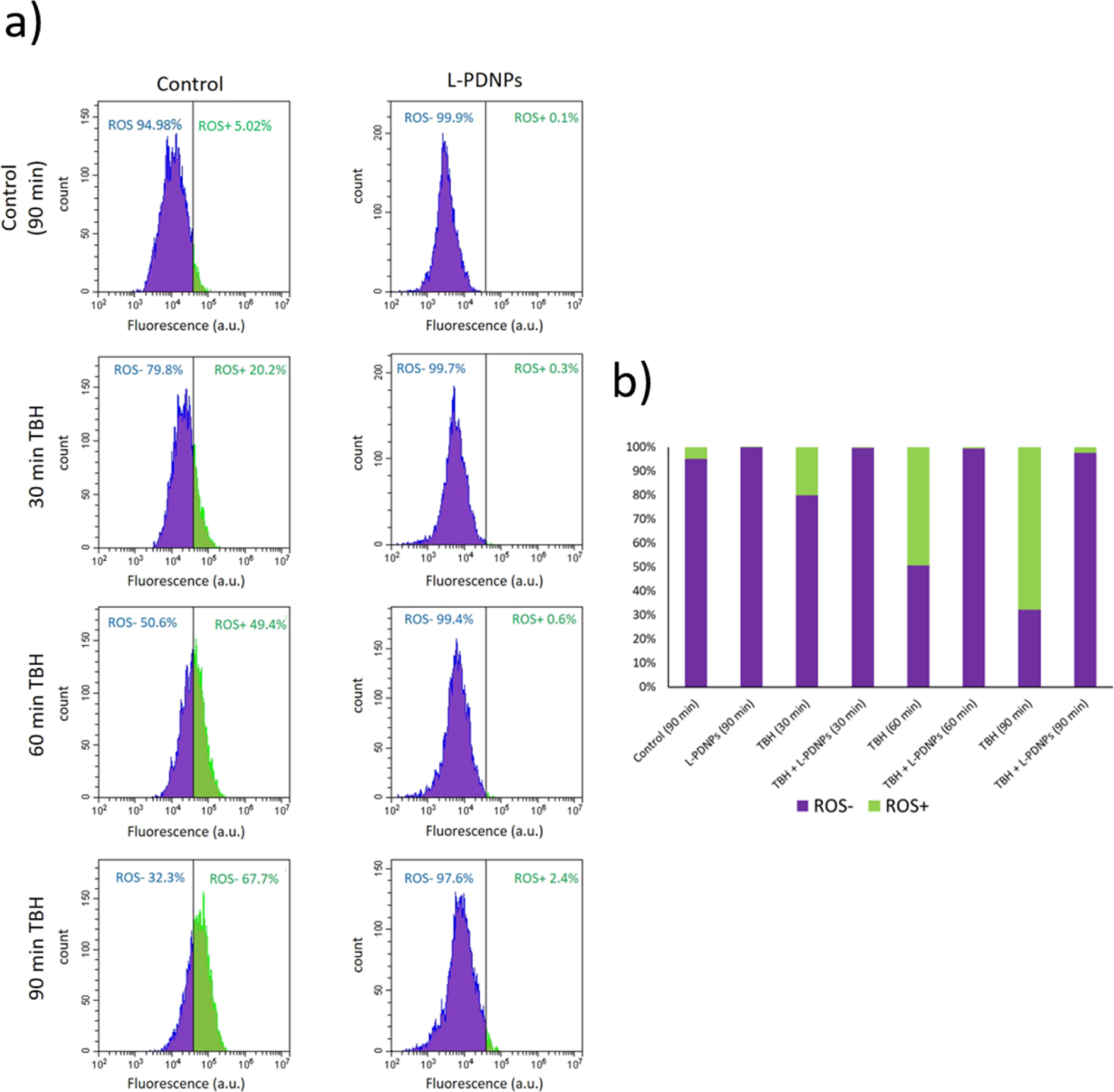
L-PDNP antioxidant effects on differentiated SH-SY5Y stained with ROS-sensitive dye CellROX. (a) Representative flow cytometry plots showing fluorescence levels of cells in different experimental conditions (in purple ROS-negative cells, ROS–; in green ROS-positive cells, ROS+). (b) Percentages of ROS+ and ROS– cells for each experimental condition (*n* = 3).

Antioxidant effects of L-PDNPs were measured using CellROX Green Reagent on differentiated SH-SY5Y with and without the treatment with 5 mM TBH, using flow cytometry. Data are shown in Figure 4; cell populations were divided into ROS negative (ROS–, purple portion of the cellular population) and in ROS positive (ROS+, green portion of the cellular population) with a fluorescence threshold based on unstained cells. As shown in Figure 4, cells without TBH treatment after 90 min from detachment showed 5.1% of ROS+ cells in the untreated control, while 0.1% of ROS+ cells were present in the sample treated with L-PDNPs. Cells treated with 5 mM TBH showed increment in ROS+ percentages with respect to the control at each measured time point (in particular 20.2% of ROS+ after 30 min of incubation with TBH, 49.4% of ROS+ after 60 min of incubation, and 67.7% of ROS+ after 90 min of incubation), while cells incubated with L-PDNPs and treated with TBH showed a lower increment in ROS+ percentages with respect to cells incubated with L-PDNPs and treated with TBH, and when compared to the corresponding time point of cells treated with just TBH (in particular 0.3% of ROS+ cells after 30 min of incubation with TBH, 0.6% of ROS+ cells after 60 min of incubation with TBH, and 2.4% of ROS+ cells after 90 min of incubation with TBH). These data show that L-PDNPs are not only able to decrease the basal level of ROS (and thus they could be used to reduce the harmful high level of oxidative stress typical of most neurological disease), but they can also almost entirely prevent the increment of ROS in the presence of pro-oxidant stimuli, thus acting as neuroprotective agents.

**Figure 5:**
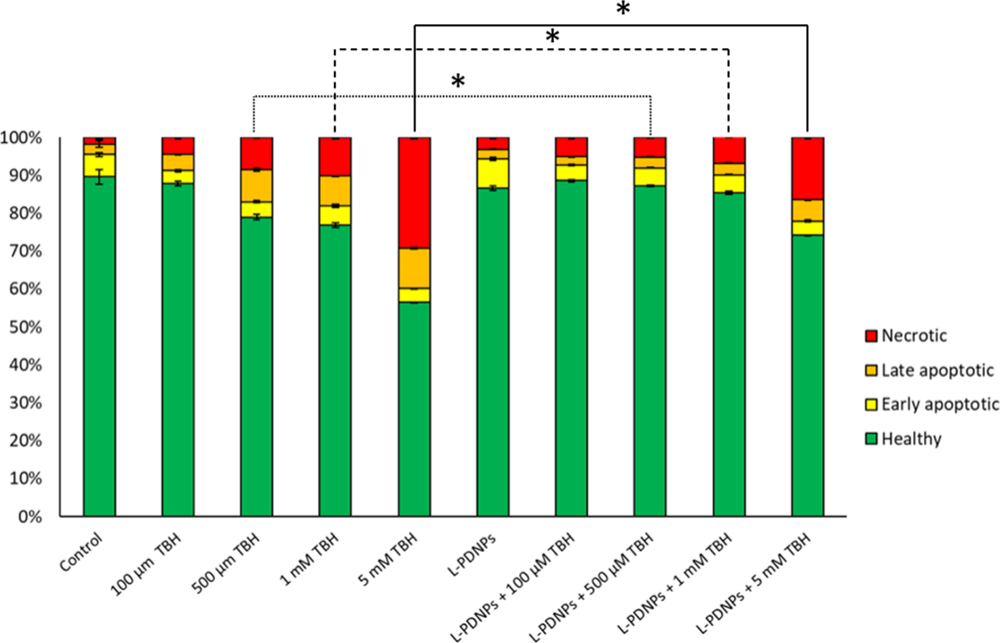
Results of flow cytometry analysis of apoptosis/necrosis levels performed on differentiated SH-SY5Y treated with or without 100 μg/mL of L-PDNPs for 72 h and incubated with different concentrations of TBH for 4 h (0 μM, 100 μM, 500 μM, 1 mM, and 5 mM). In green healthy cells, in yellow early apoptotic cells, in orange late apoptotic cells, and in red necrotic cells. (*n* = 3, *p < 0.001).

The neuroprotective effect of L-PDNPs was further confirmed by analyzing the TBH-mediated induction of apoptosis and necrosis on SH-SY5Y treated with L-PDNPs (data reported in Figures 5 and S11). After 4 h of treatment with TBH, control cells, cells treated with 100 μM TBH, cells treated with L-PDNPs, and cells treated with both L-PDNPs and 100 μM TBH did not show any statistically significant difference (*p* > 0.05) in terms of apoptosis and necrosis. However, cultures treated with 500 μM, 1 mM, and 5 mM TBH showed a statistically higher (*p* < 0.001) level of apoptotic and necrotic cells. In particular, cultures treated with 5 mM TBH showed 56.61 ± 0.13% of healthy cells, 3.61 ± 0.16% of early apoptotic cells, 10.69 ± 0.18% of late apoptotic cells, and 29.19 ± 0.42% of necrotic cells; conversely, cultures treated with both TBH and L-PDNPs showed statistically significant lower levels of apoptotic and necrotic cells: specifically, 74.19 ± 0.14% of healthy cells, 3.78 ± 0.34% of early apoptotic cells, 5.60 ± 0.09% of late apoptotic cells, and 16.42 ± 0.32% of necrotic cells. Altogether, these data strongly confirm the protective properties of L-PDNPs.

**Figure 6:**
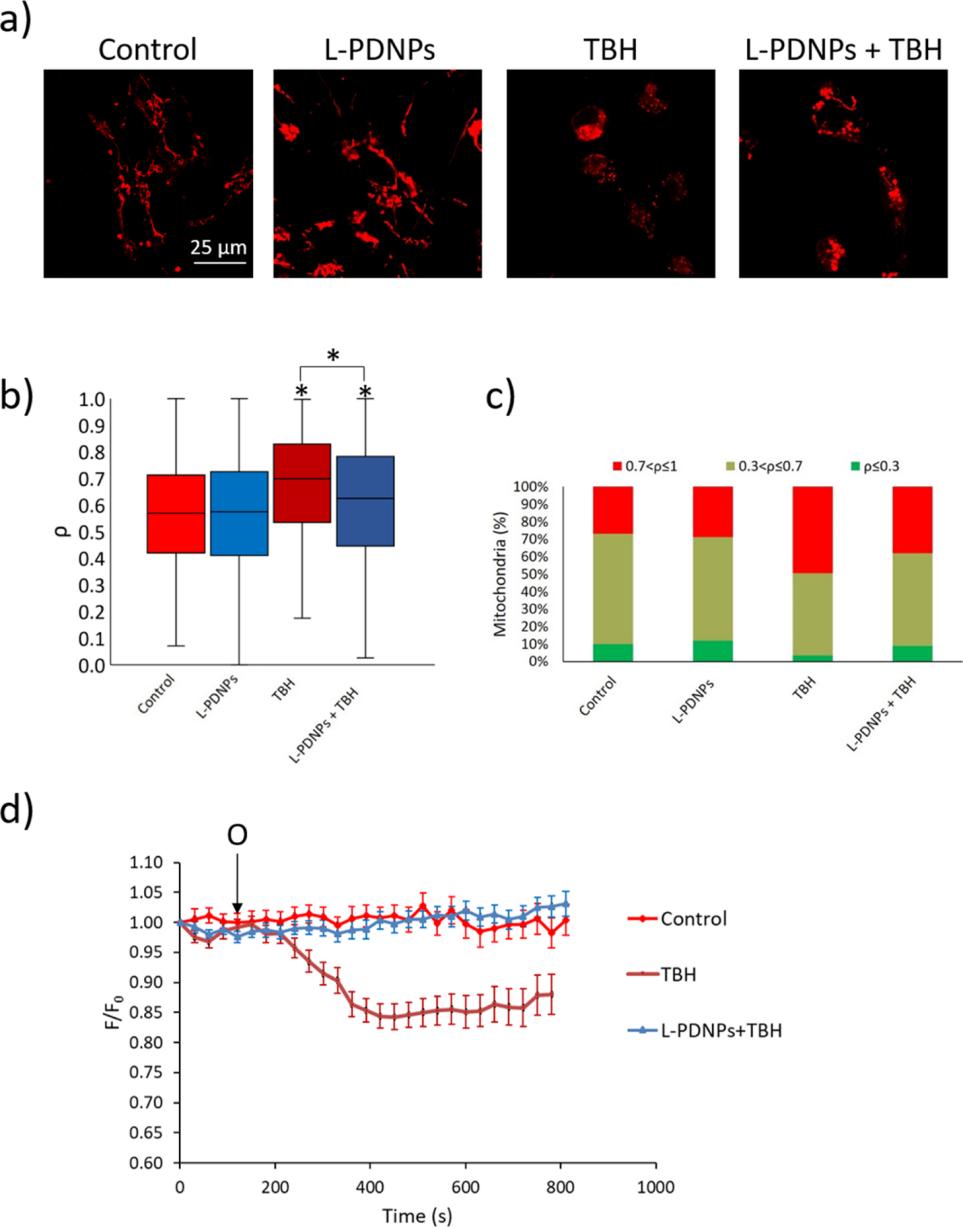
Analysis of mitochondrial morphology and membrane potential. (a) Representative images of mitochondria in different experimental conditions (mitochondria in red, stained with TMRM). (b) Median values of mitochondria roundness (ρ) in the same experimental conditions (*n* = 3, *p < 0.001). (c) Mitochondria classification in subgroups on the basis of the ρ values. (d) Trend of the mitochondrial membrane potential (ΔΨ_m_, based on fluorescence analysis). O: addition of 6 μM oligomycin (*n* = 3).

The ability of L-PDNPs to counteract mitochondrial morphology changes upon ROS over-production was assessed in differentiated SH-SY5Y. Mitochondria were classified on the basis of their roundness (ρ, calculated as described in the Material and Methods section), comparing the median ρ in different treatments (control, L-PDNPs, TBH, and L-PDNPs + TBH) and dividing the mitochondria of each experimental conditions in three different populations, namely, mitochondria with ρ ≤ 0.3, mitochondria with 0.3 < ρ ≤ 0.7, and mitochondria with 0.7 < ρ ≤ 1. Representative confocal images of each condition are shown in Figure 6a, while the analysis of mitochondrial morphology is shown in Figure 6b,c. The treatment with 100 μg/mL of L-PDNPs did not affect the morphology of mitochondria (*p* > 0.05), with the control having a median ρ of 0.57 ± 0.01 and the L-PDNPs-treated cells of 0.57 ± 0.01. Cells treated with 5 mM TBH, conversely, showed a statistically significant increment in ρ (0.70 ± 0.01). Lastly, cells treated with both 5 mM TBH and 100 μg/mL of L-PDNPs showed a statistically significant increment (*p* < 0.001) in ρ compared to both the control and the L-PDNPs treatment but also statistically lower ρ (0.62 ± 0.01; *p* < 0.001) with respect to cells treated with just TBH. This effect was reflected in the distribution of mitochondria in the various ρ classes, with the percentages of mitochondria with ρ ≤ 0.3 being 10.1% for the control, 11.8% for L-PDNPs, 3.3% for TBH, and 8.9% for L-PDNPs + TBH; the percentages of mitochondria with 0.3 < ρ ≤ 0.7 being 63.1% for the control, 59.5% for L-PDNPs, 47.3% for TBH, and 52.9% for L-PDNPs + TBH; and lastly, the percentages of mitochondria with 0.7 < ρ ≤ 1 being 26.8% for the control, 28.7% for L-PDNPs, 49.4% for TBH, and 38.1% for L-PDNPs + TBH.

It is well known how ROS can affect mitochondrial dynamics, causing alterations to the fusion and fission processes of the mitochondrial network.^33^ Several works have shown how ROS can affect mitochondria morphology in cellular models, including human fibroblasts, muscle cells, endothelial cells, and neurons.^18,34−36^ Our data show that the treatment with 5 mM TBH is able to affect the morphology of mitochondria altering their shapes, and this is reflected by higher ρ values. L-PDNPs, because of their previously discussed antioxidant and neuroprotective action on differentiated SH-SY5Y cells, are able to partially counteract this effect reducing the alterations of mitochondrial morphology caused by TBH treatment. Similar protective effects were already observed by our group by using nanoceria on H_2_O_2_-stimulated human fibroblasts.^18^

Mitochondrial protection properties were also evaluated in terms of L-PDNP ability to counteract mitochondrial membrane potential (ΔΨ_m_) collapse caused by pro-oxidant stimuli. ΔΨ_m_ collapse was evaluated with tetramethylrhodamine methyl ester (TMRM) following a procedure described in a previous work of our group.^18^ Briefly, TMRM is a cationic probe able to accumulate in polarized mitochondria. Upon ΔΨ_m_ decrement, TMRM is released by the mitochondria, causing a reduction of the fluorescence signal. The fluorescence levels of TMRM associated with SH-SY5Y were analyzed through confocal microscopy on three different experimental conditions (control, TBH, and L-PDNPs + TBH). To induce ΔΨ_m_ collapse, oligomycin (an ATP-synthase inhibitor) was administrated to the cells (“O” in Figure 6d). Control cells were able to maintain ΔΨ_m_ even after oligomycin treatment (Figure 6d, red trace), while cells treated with 5 mM TBH showed a substantial reduction in ΔΨ_m_ of 13 ± 3% after oligomycin treatment (Figure 6d, amaranth trace). Cells treated with both 5 mM TBH and 100 μg/mL of L-PDNPs (Figure 6d, blue trace) were able to maintain ΔΨ_m_ even after oligomycin treatment.

**Figure 7:**
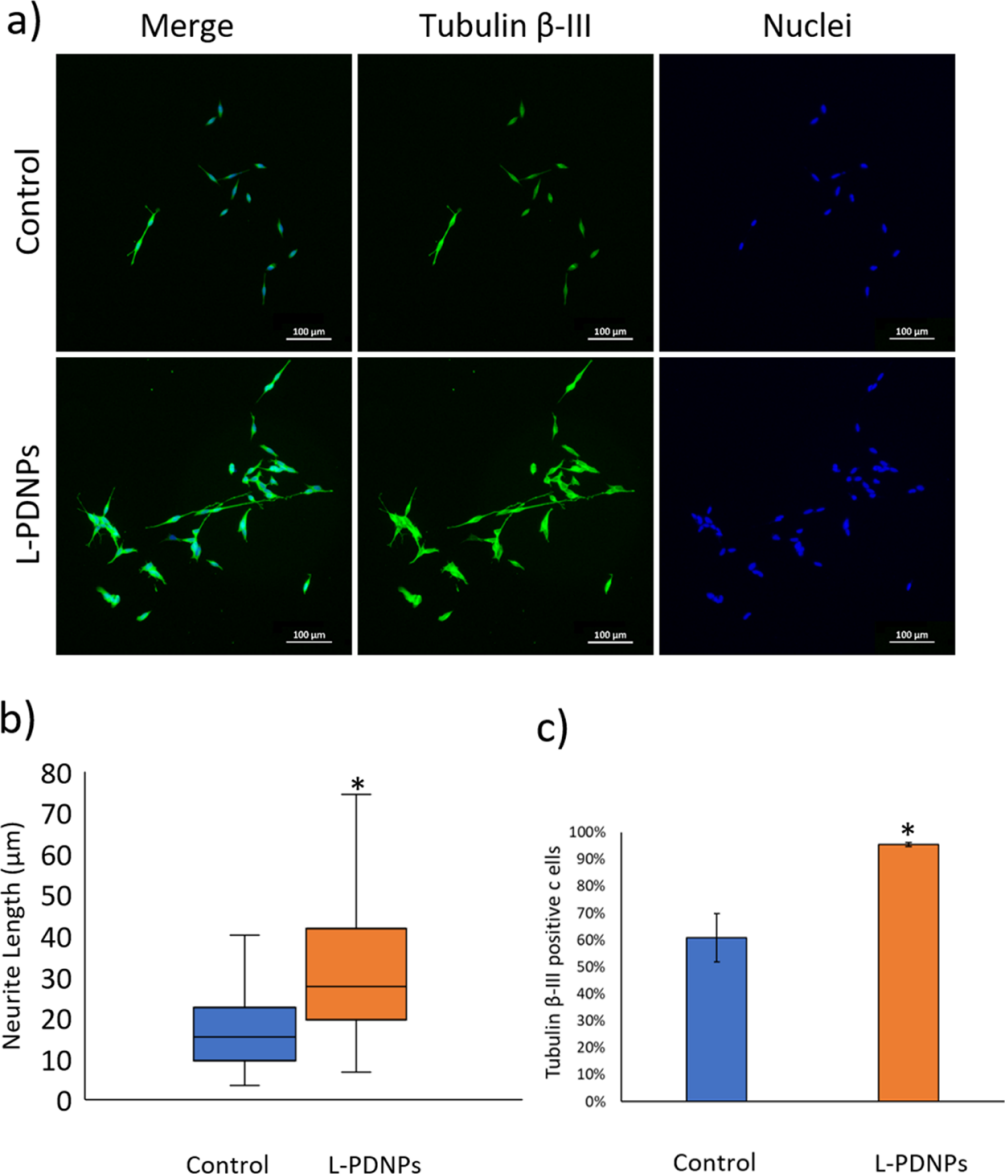
Analysis of the L-PDNP effect on neurite outgrowth of differentiating SH-SY5Y cells. (a) Representative epifluorescence images of SH-SY5Y treated or not with L-PDNPs (tubulin β-III in green, nuclei in blue). (b) Comparison of the median neurite length of SH-SY5Y with or without L-PDNP treatment (*n* = 3, *p < 0.001). (c) Percentages of tubulin β-III-positive cells with and without L-PDNP treatment (*n* = 3, *p < 0.001).

L-PDNP ability to stimulate neurite outgrowth and neuronal differentiation in SH-SY5Y cells was assessed through epifluorescence microscopy (Figure 7a). After three days of incubation with differentiation medium, SH-SY5Y showed a median length of neurite of 15.4 ± 0.7 μm, while cells treated for 72 h with the same medium doped with 100 μg/mL of L-PDNPs showed a statistically significant (*p* < 0.001) increment in neurite length, with a median neurite length of 27.5 ± 1.2 μm (Figure 7b, blue and orange boxes, respectively). The ability of L-PDNPs to stimulate neuronal differentiation was further confirmed by the analysis of the expression of tubulin β-III; in particular, it was observed that the treatment with 100 μg/mL of L-PDNPs was able to significantly (*p* < 0.001) increase the percentage of tubulin β-III-positive cells with respect to controls (tubulin β-III positive cells 60.9 ± 9.0% in the case of control cultures and 95.5 ± 0.7% in the case of treated cultures, Figure 7c). Other antioxidant nanostructures have shown similar effects upon neuronal cells: our group, for example, showed how various formulation of nanoceria could stimulate neurite outgrowth in both SH-SY5Y and PC12 cells.^15,16,19^ Other groups reported that polydopamine-coated substrates have a positive effect on neuronal cell attachment, viability, and neuronal differentiation.^37,38^ We hypothesize that the antioxidant properties of L-PDNPs are at the basis of this neuronal differentiation enhancement phenomenon: it is well known that ROS are involved in cell differentiation, and ROS levels can affect the differentiative destiny of various cell types.^39,40^ The L-PDNP ability to change the SH-SY5Y ROS level can shift them toward a more favorable condition for a differentiation pathway, opening new interesting perspectives in neuronal regeneration applications.

### NIR Photothermal Conversion and Cell Stimulation

NIR absorption of L-PDNPs was first assessed through spectrophotometric measurements, and a concentration-dependent increment behavior was observed (Figure S12a). In particular, the absorption at 808 nm (the wavelength of the laser used in the following experiments) was 0.101 a.u. for 30 μg/mL of L-PDNPs, 0.230 a.u. for 62.75 μg/mL, 0.470 a.u. for 125 μg/mL, and 0.980 a.u. for 250 μg/mL (Figure S12b). The heating generated by NIR irradiation was evaluated using a thermocouple; in particular, after 60 s of irradiation with a 532 mW laser power and a 2.5 mm spot size, we observed an increment in temperature of 34.5 °C using a dispersion of L-PDNPs of 5 mg/mL, an increment of 31.5 °C with a dispersion of 1 mg/mL, an increment of 24 °C with a dispersion of 500 μg/mL, an increment of 22.5 °C with a dispersion of 250 μg/mL, and an increment of 16 °C with a dispersion of 100 μg/mL, while without L-PDNPs no significant temperature increment was observed upon NIR irradiation (Figure S12c).

**Figure 8:**
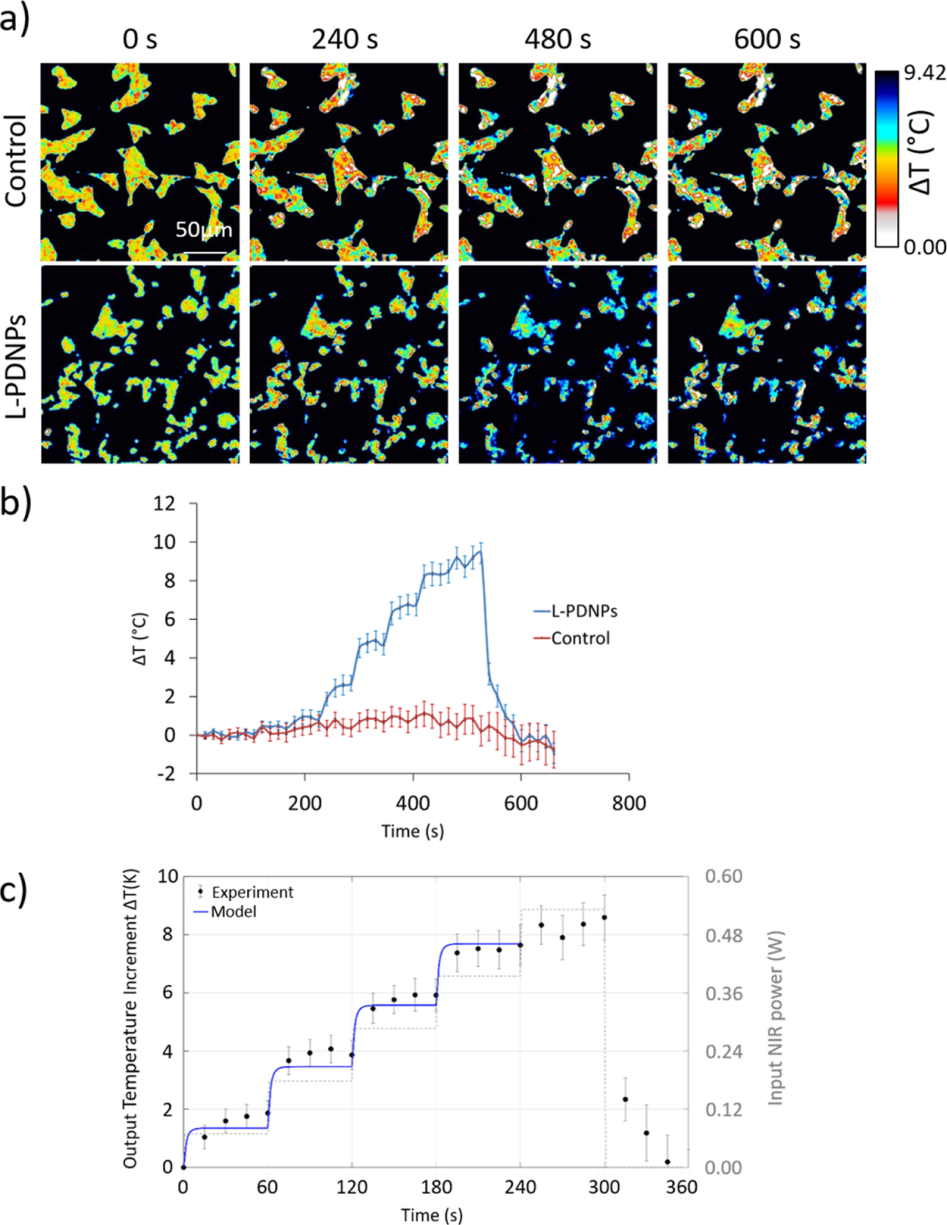
Analysis of intracellular temperature dynamics in SH-SY5Y cells during NIR stimulation with and without L-PDNPs. (a) Representative time frames before (*t* = 0), during (*t* = 240 and 480 s), and after (*t* = 600 s) NIR stimulation. Temperature changes (Δ*T*) from the baseline (25.0 ± 1.0 °C) are represented as pseudo-colors. (b) Δ*T* (°C) during the NIR stimulation (*n* = 3). (c) Temperature increment (left *y*-scale) *versus* time at the center of the NIR irradiation spot, as caused by the chosen time law for the input NIR power (right *y*-scale). Experimental measurements (circles with vertical bars to represent mean ± standard error, *n* = 3) are shown, together with the theoretical trend (solid curve) provided by the developed model.

The intracellular temperature increment was quantified through confocal microscopy imaging using the ER Thermo Yellow temperature-sensitive fluorescent dye (representative frames shown in Figure 8a). As shown in Figure 8a, irradiation with NIR laser in control cells did not cause any decrement of cell fluorescent levels, corresponding to no temperature increment (red trace in Figure 8b). Treatment with NIR laser at low powers (0.148, 0.425, and 1.000 mW) did not cause any decrement in the fluorescence level even in cells treated with L-PDPNs, corresponding to no temperature increment in intracellular temperature (blue trace in Figure 8b). An increment of the intracellular temperature was observed with 69.5 mW of laser power (2.69 ± 0.37 °C). Laser power was increased every 60 s causing additional temperature increments at each power step (Δ*T* of 4.70 ± 0.68 °C was observed with 178 mW laser power, 6.70 ± 0.94 °C with 286.5 mW, 8.47 ± 0.81 °C with 395 mW, and 9.40 ± 0.20 °C with 532 mW). After reaching the maximum laser power output (532 mW), the NIR laser was turned off, and this caused a recovery to the basal conditions of the fluorescence levels, and therefore of the temperature, in the cells. These results indicate that the combination of L-PDNPs and NIR stimulation can be exploited to produce localized heating of neuronal cells. In particular, by changing the laser power output, a fine-tuning of intracellular temperature can be achieved. This experiment is the first analysis of the NIR-induced heating of a polydopamine-based nanomaterial at the cellular level by means of a thermosensitive dye, and it also represents, to the best of our knowledge, the first demonstration of an organic antioxidant nanostructure achieving a precise and localized tuning of cellular temperature. The controlled alteration of the intracellular temperature is an interesting tool for plenty of applications, including cancer treatment, muscle activation, and induction of cellular differentiation;^30,41,42^ however, the vast majority of approaches in the literature make use of inorganic materials. Our work instead presents a fine characterization of the photothermal conversion abilities of L-PDNPs and of their effects at the cellular level, which opens possibilities to new and interesting applications of dopamine-based nanomaterials even beyond neuronal stimulation, owing to their organic and biodegradable nature.

Collected experimental data were also used for calibrating a theoretical model able to describe the considered temperature variation because of NIR stimulation. Temperature increment associated with NIR irradiation of SH-SY5Y pre-incubated with L-PDNPs is proposed again in Figure 8c, as caused by the chosen time law for the input NIR power. In particular, experimental measurements taken at the center of the NIR irradiation spot are shown, together with the trend provided by a theoretical model based on the following differential problem
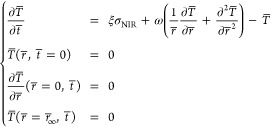
1where *T̅* = *T̅*(*r̅*, *t̅*) denotes the unknown temperature increment, as a function of space (*r̅*) and time (*t̅*) variables, σ_NIR_ = σ_NIR_(*r̅*, *t̅*) represents the nominal NIR intensity, ξ is a calibration parameter accounting for the NIR intensity fraction contributing as heat source, *r̅*_∞_ defines the upper extreme of the spatial range, and ω accounts for physical parameters relevant to heat conduction and convection. Additional details, as well as parameter values, are reported in the Material and Methods section, for ease of presentation. With reference to Figure 8c, experimental data show that temperature increments accurately followed the input power steps, with a damped reaction only to the last step (at 240 s), most probably because of saturation phenomena (in light of this issue, model integration was advanced up to 240 s). Moreover, the reasonably good agreement between experimental data and theoretical trend (RMSE = 0.33 K) suggests the possibility to suitably characterize and control the induced thermal effect, as needed when also considering safety thresholds. Stronger claims, however, should be based on further investigations on complex *in vivo* application domains.

**Figure 9:**
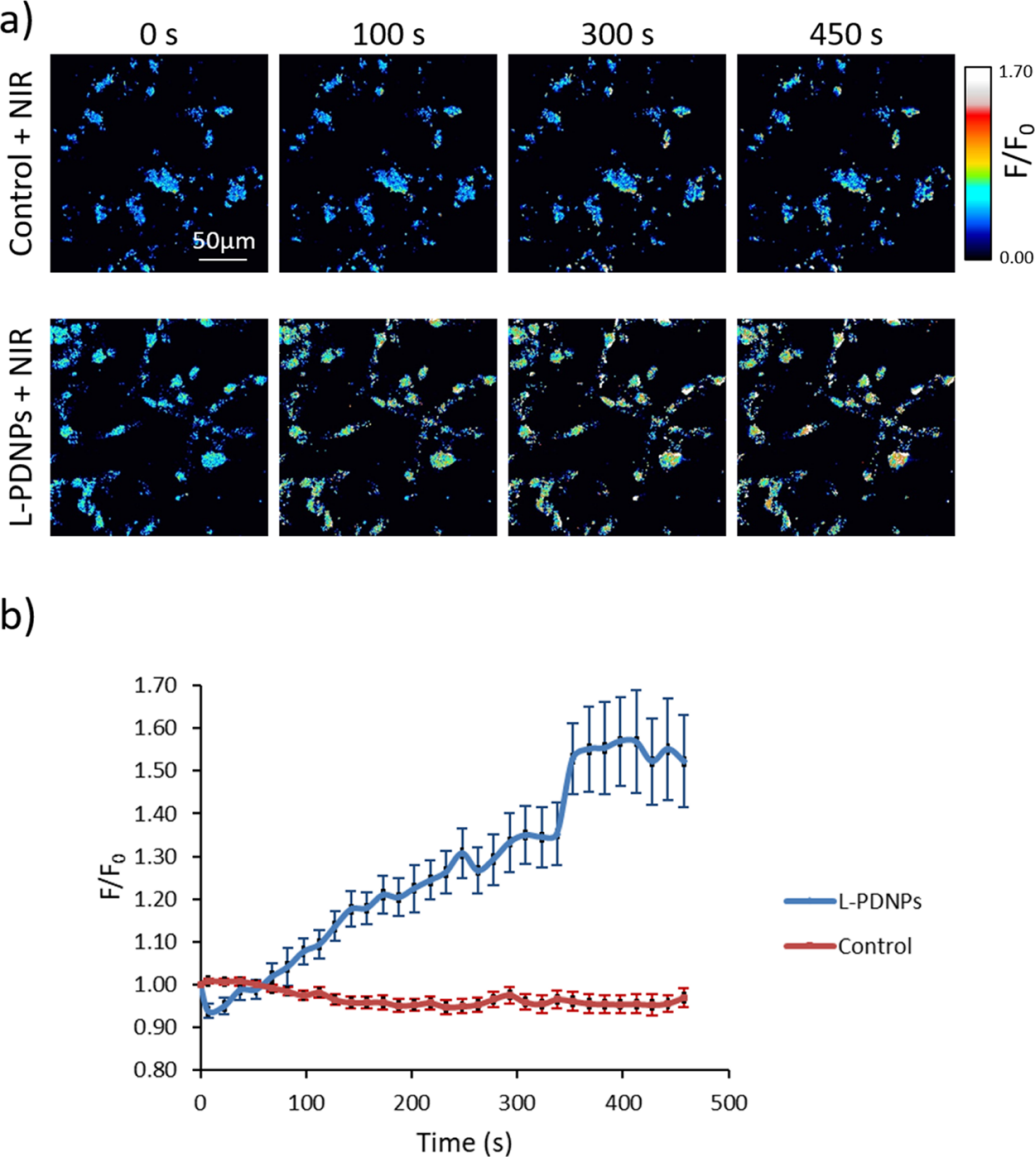
(a) Representative time frames of calcium imaging performed on differentiated SH-SY5Y cells in the presence of NIR laser stimulation with and without L-PDNPs. (b) Time course of the variation of cell fluorescence levels, indicative of calcium concentration, during NIR stimulation in both experimental conditions (*n* = 3).

The ability of L-PDPNs to stimulate neuronal activation upon NIR irradiation was eventually investigated performing calcium imaging. As shown in Figure 9a, the stimulation of control cultures nontreated with L-PDNPs did not cause any change in the fluorescence levels measured with Fluo-4 AM, thus meaning no increment of the Ca^2+^ intracellular level (Figure 9b, red trace). On the other hand, the stimulation of L-PDNPs-treated SH-SY5Y cells (Figure 9b, blue trace) with NIR laser caused a continuous and constant increment in the fluorescence (up to approximately 50% over 450 s of irradiation), translatable into an increment of the intracellular Ca^2+^ levels. The presence of an intracellular Ca^2+^ increment only in the presence of L-PDNPs and NIR stimulation indicates that photoinduced effects of NIR laser are not at the basis of the observed cellular activation, yet this phenomenon is because of the photothermal conversion effect of L-PDNPs and to the consequent increment of the cellular temperature.

Finally, we also investigated the effect of NIR stimulation on the ROS production in SH-SY5Y cells (Figure S13). In particular, we analyzed the ROS levels of control cells, of cells treated with L-PDNPs, of cells stimulated with NIR radiation (same parameters in terms of laser power and exposure time previously described), and of cells treated with L-PDNPs and stimulated with NIR radiation. No statistically significant differences (*p* > 0.05) in terms of the ROS level among the various conditions were observed.

Nanoparticle-mediated stimulation of neuronal cells is already being investigated: our group, as an example, carried out pioneering studies on the use of piezoelectric nanomaterials for the wireless stimulation of neuronal cells.^43,44^ Other groups reported the use of NIR-responsive nanomaterials for the remote stimulation of neuronal function: for example, NIR-responsive gold nanorods have been exploited to stimulate primary auditory neurons.^45^ NIR-responsive nanomaterials have also been proposed as a possible countermeasure against neurological disease hallmarks, with studies reporting the use of NIR-responsive gold nanorods as a tool to prevent the formation of amyloid-beta (Aβ) fibrils and to dissolve already formed aggregates typical of Alzheimer’s disease.^46,47^ Despite the impressive results, all these approaches involve the use of inorganic nanomaterials, presenting the previously discussed drawback caused by a low degradability of such materials in a biological environment.

Our results demonstrate that L-PDNPs can be used as a remote tool to activate and elicit neuronal functions, and represent, to date, the first and only example of a completely organic nanostructure exploitable in NIR-mediated neuronal stimulation. Of course, further investigations involving *in vivo* models are of pivotal importance for future exploitations of polydopamine-based nanomaterials in neuronal stimulation procedures; however, the results obtained in this work are, in our opinion, promising enough to justify efforts in this direction.

## Conclusions

L-PDNPs demonstrated the ability to stimulate differentiation of SH-SY5Y cells and to protect neuron-like cells from ROS-induced damages, reducing ROS accumulation caused by treatment with pro-oxidant stimuli, and counteracting ROS-induced alteration in mitochondrial morphology. We also reported the possibility of using L-PDNPs as a photoconversion agent able to tune the intracellular temperature of SH-SY5Y upon NIR laser stimulation. Moreover, we showed the possibility to suitably characterize and control the induced thermal effect with a theoretical model of L-PDNPs-based NIR-mediated heating, calibrated by means of data obtained with a thermosensitive dye. This is the first time that a fully organic and biodegradable nanotechnological antioxidant has been shown able to elicit neuronal activity upon NIR irradiation. Taken all together, these properties describe L-PDNPs (and polydopamine-based nanomaterials in general) as promising multitasking tools able to produce beneficial effects on neuronal cells and to tune their cellular activity, providing a potentially disruptive instrument for the treatment of neurological diseases.

## Materials and Methods

### mPEG-DSPE-Coated PDNPs Synthesis

The PDNP Synthesis procedure was adapted from Bao *et al.*,^23^ and consisted of a classic Stöber method. Briefly, 90 mL of Milli-Q water, 40 mL of ethanol, and 2 mL of ammonium hydroxide solution (Sigma-Aldrich) were mixed for 30 min under stirring; 500 mg of dopamine hydrochloride (Sigma, in 10 mL Milli-Q water) was then added to the mixture, and the reaction was left under stirring overnight. Afterward, the mixture was diluted 1:1 in ethanol at 4 °C and centrifuged at 8960*g* for 30 min at 4 °C. After the centrifuge, the supernatant was discarded, and the pellet was resuspended in Milli-Q water and washed three times at 8960*g*. After the cleaning, the obtained PDNPs were quantified through freeze-drying and weighting. PDNPs were functionalized with mPEG-DSPE (5000 Da, Nanocs) through sonication: briefly, 20 mg of PDNPs and 20 mg of mPEG-DSPE were suspended in 3 mL of Milli-Q water and sonicated using a tip sonicator (Thermo Fisher, Fisher Scientific FB120) for 15 min at 50% power. After sonication, the obtained L-PDNPs were washed three times by centrifugation at 16602*g* for 15 min at room temperature.

### Electron Microscopy

Particle morphology and size were determined through SEM analysis. Briefly, 5 μL of a suspension of 100 μg/mL of PDNPs was drop-cast on a small piece of a silicon wafer and let dry. After the drop dried, the sample was gold-sputtered using a Quorum Tech Q150RES Gold Sputter Coater with 30 mA for 60 s and imaged using an SEM system, Helios NanoLab 600i FIB/SEM, FEI.

Bright-field TEM images were acquired by a JEOL JEM-1400Plus TEM, with a thermionic source (LaB6), operated at 120 kV. For TEM analyses, 10 μL of the sample suspensions were drop-cast onto an ultrathin carbon-coated 150 mesh copper grid.

### DLS Measurements

DLS (Malvern-Zetasizer Nano ZS90) was used to determine the average hydrodynamic diameter, *Z*-potential, and PDI of L-PDNPs; 100 μg/mL of L-PDNPs in Milli-Q water was used for all the measurements using disposable polystyrene cuvettes (Malvern Zetasizer Nano series) to measure the hydrodynamic diameter and disposable folded capillary cells (Malvern Zetasizer Nano series) for measuring surface *Z*-potential.

### TGA and Porosity Analysis

TGA was carried out with a TGA Q500-TA Instrument. During TGA, samples were heated from 30 to 600 °C at a heating rate of 5 °C/min under a nitrogen atmosphere set at a flow rate of 50 mL/min. PDNPs, mPEG-DSPE, and L-PDNPs behaviors were analyzed to assess the percentage in weight of mPEG-DSPE coating the PDNPs.

Nitrogen physisorption measurements were carried out at 77 K using a gas sorption analyzer, model Autosorb-iQ (Quantachrome Instruments). The samples were initially degassed for 3 h at 90 °C under vacuum conditions to remove weakly adsorbed species. The specific surface areas were calculated by using the multipoint BET model, considering six equally spaced points in the *P*/*P*_0_ range from 0.05 to 0.30. The pore size distribution was determined from the desorption isotherms (range 0.35 < *P*/*P*_0_ < 1.00) utilizing the Barrett–Joyner–Halenda method.

### EPR Spectroscopy

The antioxidant activity of PDNPs and L-PDNPs was studied by EPR spectroscopy coupled with the spin-trapping technique. Hydroxyl radicals were generated *in situ* by Fenton reaction and trapped using the spin-trap DMPO (Sigma). H_2_O_2_ (20 μL, 10 mM), DMPO (100 μL, 50 mM), an aqueous dispersion of nanoparticles at different concentrations (78 μL), and FeSO_4_·7H_2_O (2 μL, 10 mM) were mixed in an Eppendorf tube. The resulting solution was mixed, transferred to a quartz microcapillary tube, and placed in the EPR cavity for measurement. After 5 min since the addition of FeSO_4_·7H_2_O, the spectra were recorded on a Bruker EMXnano X-Band spectrometer (Bruker). The EPR measurement conditions were as follows: frequency 9.74 GHz, scan width 100 G, receiver gain 60 dB, time constant 1.28 ms, sweep time 80 s, scan 1. After the acquisition, the spectra were processed using the Bruker Xenon software (Bruker) for baseline correction, and the total number of hydroxyl radicals trapped was quantified using the SpinFit software (Bruker).

### L-PDNP Degradation Analysis

The degradation profile of L-PDNPs was qualitatively studied in buffer solutions of pH 7.4 and 4.5, with or without the addition of 1 or 10 mM of H_2_O_2_. Briefly, 500 μg/mL of nanoparticles were dispersed at a final volume of 1 mL for each condition and were shaken at 37 °C for up to 15 days. At predetermined time points (0, 1, 7, and 15 days), 20 μL of each dispersion were cast on a silicon wafer surface, attached on an SEM stub, and let to dry. The dried samples were gold-sputtered with a Quorum Tech Q150RES Gold Sputter Coater at 30 mA for 60 s and studied using a SEM system (Helios NanoLab 600i FIB/SEM, FEI).

### Cell Culture and Biocompatibility Assessment

For all the studies involving cell culturing presented in this work, differentiated SH-SY5Y human neuroblastoma cells (ATCC CRL-2266) were used. SH-SY5Y were cultured in proliferation condition using Dulbecco’s modified Eagle’s medium F-12 (DMEM, Sigma, with 15 mM HEPES) supplemented with 10% heat-inactivated fetal bovine serum (FBS, Gibco), 1% l-glutamine (stock 200 mM, Gibco), 1% sodium pyruvate (stock 100 mM, Gibco), and 1% penicillin–streptomycin (100 IU/mL of penicillin and 100 μg/mL of streptomycin, Gibco). Cells were differentiated by substituting proliferative medium 24 h after seeding with DMEM high glucose (Sigma-Aldrich) supplemented with 1% heat-inactivated FBS (Gibco), 1% l-glutamine (stock 200 mM, Gibco), 1% sodium pyruvate (stock 100 mM, Gibco), 1% penicillin–streptomycin (100 IU/mL of penicillin and 100 μg/mL of streptomycin, Gibco), and all-*trans*-retinoic acid (Sigma, 10 μM). For splitting and cell detachment procedures, the cell culture medium was removed, cells were washed with Dulbecco’s phosphate-buffered saline (DPBS, Sigma), and incubated 5 min with trypsin (Sigma).

L-PDNP effects on SH-SY5Y viability was assessed through the Quant-iT PicoGreen dsDNA Assay Kit (Invitrogen). Briefly, SH-SY5Y were seeded in a 48-well plate (Corning) at 10,000 cells/cm^2^ density and let to adhere for 24 h in proliferation medium. After 24 h, the cells were put in differentiation medium and left to differentiate for 72 h. Thereafter, cultures were incubated with fresh differentiation medium containing different concentrations of L-PDNPs (0, 31.25, 62.5, 125, and 250 μg/mL) and left in incubation for either 24 or 72 h. After the incubation with L-PDNPs, the cells were washed in DPBS (Sigma), suspended in 100 μL of Milli-Q water, and subjected to three cycles of freezing/thawing (from −80 to 37 °C) to allow cell lysis and dsDNA release. Quant-iT PicoGreen dsDNA assay was carried out mixing cell lysate, PicoGreen reagent, and buffer in Corning Costar 96-well black polystyrene plates following the manufacturer’s instructions. Fluorescence (excitation 485 nm, emission 535 nm) was measured with a Victor X3 Plate Reader (Perkin Elmer).

L-PDNP biocompatibility was also assessed using a LIVE/DEAD cell viability assay (Thermo Fisher). SH-SY5Y were seeded in 24-well plates (Corning) at 10,000 cells/cm^2^ and treated as previously described (24 h in proliferation medium, 72 h in differentiation medium, and then 72 h in differentiation medium supplemented with different concentrations of L-PDNPs (in particular, 0, 31.25, 62.5, 125, and 250 μg/mL). After the incubation with L-PDNPs, cells were washed with DPBS and incubated with phenol red-free differentiation medium supplemented with 5 μg/mL of Hoechst (Invitrogen), 4 μM of ethidium homodimer-1, and 2 μM of calcein-AM for 20 min (all reagents from Thermo Fisher). After the staining, cells were washed with DPBS and imaged using fluorescence microscopy (Eclipse Ti, Nikon) with a 10× objective. Obtained images were analyzed using ImageJ by counting relative numbers of dead cells (ethidium homodimer-1-positive cells) and live cells (calcein-positive cells) in each condition.

### Nanoparticle–Cell Interaction Investigations

The ability of L-PDNPs to be internalized by SH-SY5Y was first assessed by confocal microscopy. At this aim, L-PDNPs were stained with DiO dye (Vybrant Multicolor Cell-Labeling Kit, Thermo Fisher scientific): briefly, 20 μM of DiO was added to 1 mL of water containing 10 mg/mL of L-PDNPs, and the suspension was left under agitation for 2 h; thereafter, the nanoparticles have been washed three times with water through centrifugation at 16602*g*.

For confocal-based analysis, SH-SY5Y were seeded at 10,000 cells/cm^2^ in μ-Plate 24-well Black (Ibidi), left attaching 24 h in proliferation medium, and subsequently put in differentiation medium for 72 h. Thereafter, the cells were incubated with fresh differentiation medium containing 100 μg/mL of DiO-stained L-PDNPs. Cultures were then fixed with 4% paraformaldehyde (PFA, Sigma, in DPBS) at 4 °C for 20 min and subsequently washed twice with DPBS (Sigma) at different time points from the incubation (30 min, 4 h, 24 h and 72 h). For internalization analysis, the cytoskeleton of fixed cells was stained with TRITC-phalloidin (Sigma), and the nuclei were marked with Hoechst (Invitrogen). Cells were incubated 40 min with 10% goat serum (Sigma) in DPBS (Sigma) and subsequentially with a solution of 10% goat serum containing 2.5 μg/mL of TRITC-phalloidin (Sigma) and 5 μg/mL of Hoechst (Invitrogen) for 1 h; after the incubation, cells were washed twice in DPBS (Sigma) and then imaged with a confocal microscope (C2s system, Nikon) with a 60× oil immersion objective. Both 2D images and 3D rendering were acquired for each condition.

For internalization analysis through flow cytometry, SH-SY5Y were seeded at 10,000 cells/cm^2^ in 24-well plates (Corning) and left in proliferation medium for 24 h; thereafter, cells were incubated with differentiation medium for 72 h and, after three days, further incubated with differentiation medium containing 100 μg/mL of DiO-stained L-PDNPs. At different time points (30 min, 4 h, 24 h, and 72 h), cells were detached from the multiwell plate, and their fluorescence intensity (excitation 488 nm, emission 525 ± 40 nm) was assessed through flow cytometry (CytoFLEX platform, Beckman Coulter) and compared to the fluorescence level of untreated SH-SY5Y cells. Data were analyzed with the software CytExpert (Beckman Coulter).

For SEM analysis, SH-SY5Y cells were seeded at 10,000 cells/cm^2^ on Willco Petri dishes (GWST-3512) and incubated in proliferation medium for 24 h. After 24 h, the cells were incubated with differentiation medium for 72 h and consequently incubated for further 72 h with 100 μg/mL of L-PDNPs in differentiation medium. After the incubation with L-PDNPs, cells were fixed in PFA as previously mentioned, washed with Milli-Q water, and double-fixed with glutaraldehyde 2.5% in Milli-Q water for 2 h at 4 °C. After the second fixation procedure, cells were dehydrated with increasing ethanol concentrations (25, 50, 75, and 100% in water, incubation of 5 min for each condition), dried, gold-sputtered using a Quorum Tech Q150RES Gold Sputter Coater at 30 mA for 60 s, and eventually imaged with a SEM system (Helios NanoLab 600i FIB/SEM, FEI).

To assess the intracellular fate of L-PDNPs, cells were seeded at 10,000 cells/cm^2^ in Willco Petri dishes (GWST-3512) and treated as previously described (24 h in proliferation medium, 72 h in differentiation medium, and finally incubated with DiO-L-PDNPs for different times; in particular, 30 min, 4 h, 24 h, and 72 h). After the incubation with DiO-L-PDNPs, cells were rinsed with DPBS and incubated with phenol red-free differentiation medium supplemented with 5 μg/mL of Hoechst and either with 1 μM of LysoTracker Red (Thermo Fisher) or with 1 μM of tetramethyl rhodamine methyl ester (TMRM, Life Technologies) for 30 min. After the incubation, cells were rinsed with DPBS and then imaged with a confocal microscope (C2s system, Nikon) with a 60× oil immersion objective. The co-localization between DiO-L-PDNPs and either lysosomes or mitochondria was analyzed using NIS-elements software (Nikon) by measuring the Pearson correlation coefficient.

### BBB *In Vitro* Model and L-PDNP Crossing Abilities

In order to assess the abilities of L-PDNPs to cross BBB and to reach the brain environment, an *in vitro* model of the BBB based on transwell inserts was developed. Brain endothelial cells (bEnd.3, ATCC CRL-2299) cultured in DMEM high glucose (Sigma-Aldrich) supplemented with 10% heat-inactivated FBS, 2 mM l-glutamine, 1 mM sodium pyruvate, 100 IU/mL of penicillin, and 100 μg/mL of streptomycin (all from Gibco) were used. bEnd.3 cells were seeded at 30,000 cells/cm^2^ density on porous cell culture inserts made of transparent poly(ethylene terephthalate) membranes inserted in 24-well plates (inserts provided by Falcon, pores size 3 μm). bEnd.3 cells were grown for 5 days, and then, the system was characterized and exploited for permeability studies. Bioelectrical properties of the BBB model were assessed by measuring the TEER with a Millipore Millicell ERS-2 Volt-Ohmmeter. BBB integrity was verified by measuring the passage of FITC-dextran (70 kDa, Sigma) at different time points. Fresh medium (500 μL) was added on the barrier abluminal compartments, and 200 μL of 200 μg/mL FITC-dextran solution were added in the barrier apical compartments (on the top; solutions were prepared in phenol red-free complete medium). Membranes without cells were considered as a control. Analyses were conducted by measuring the fluorescence (excitation 485 nm, emission 535 nm) of medium recovered in the abluminal compartment at 24 and 72 h with a Victor X3 Plate Reader (PerkinElmer).

The formation of tight junctions was assessed through immunostaining against the ZO-1 protein. Cells, after 5 days of culture, were fixed with 4% PFA for 20 min at 4 °C and permeabilized with Triton 0.1% X-100 for 15 min; afterward, cultures were blocked with 10% of goat serum for 1 h and incubated with primary antibody anti-ZO-1 (2.5 μg/mL, Abcam) for 3 h at room temperature. Cells were washed three times in DPBS and incubated with goat serum 10% supplemented with 10 μg/mL of F(ab′)2-goat anti-Rabbit IgG (H + L) Alexa Fluor 488 conjugate (Invitrogen), 2.5 μg/mL of TRITC-phalloidin (Sigma), and 5 μg/mL of Hoechst (Invitrogen) at 37 °C for 45 min. After three DPBS rinsing steps, images were acquired by a confocal microscope (C2s, Nikon) using a 60× oil immersion objective.

After the model characterization, the ability of L-PDNPs to cross the bEnd.3 cell monolayer was assessed. Transwell inserts seeded with 30,000 cells/cm^2^ were let grown for 5 days; thereafter, fresh medium was added to each well: in particular, 500 μL of medium were added in the basolateral side of the insert and 200 μL of medium supplemented with 50 μg of L-PDNPs were added in the apical side of the insert (phenol red-free fully complemented media were used). After 72 h, the medium in the basolateral compartment of the transwell inserts was collected and the absorbance was measured using a Victor X3 Plate Reader (PerkinElmer), in order to evaluate the concentration of L-PDNPs in the basolateral compartment (by exploiting a calibration curve obtained at 490 nm). To assess the internalization of the particles by the bEnd.3 monolayer, transwell inserts were treated as previously described with 50 μg/mL of DiO-L-PDNPs for 72 h. After 72 h, cells were fixed with 4% PFA and stained with Hoechst and TRITC-phalloidin as previously described and imaged with a confocal microscope (C2s, Nikon) using a 60× oil immersion objective.

### Assessment of L-PDNP Antioxidant Activity on Neuronal Cells

For the evaluation of the antioxidant effects of L-PDNPs on SH-SY5Y, 10,000 cells/cm^2^ were seeded into 24-well plates (Corning) and incubated as previously described (24 h in proliferation medium, 72 h in differentiation medium, and further 72 h in differentiation medium doped with 100 μg/mL of L-PDNPs). After the incubation with L-PDNPs, cells were stained with CellROX Green Reagent (Invitrogen) at 2.5 μM for 30 min in differentiation medium and subsequently detached. Half of the samples were treated with 5 mM Luperox TBH70X, TBH solution (Sigma-Aldrich). The relative fluorescence intensity of all of the experimental conditions (control, cells treated with TBH, cells treated with L-PDNPs, and cells treated with L-PDNPs and TBH) was measured by flow cytometry (excitation 488 nm, emission 525 ± 40 nm) at different time points from the addition of TBH (30, 60, and 90 min). Data were analyzed with the software CytExpert (Beckman Coulter).

To analyze the protective effect of L-PDNPs, SH-SY5Y were seeded at 10,000 cells/cm^2^ into 24-well plates (Corning) and incubated as previously described (24 h in proliferation medium, 72 h in differentiation medium, and further 72 h in differentiation medium doped with 100 μg/mL of L-PDNPs). Thereafter, cells were rinsed with PBS and incubated in differentiation media supplemented with various concentrations of TBH (0 μM, 100 μM, 500 μM, 1 mM, and 5 mM) for 4 h. After the treatment with TBH, cells were rinsed with PBS, detached, and stained with Annexin V-FITC/propidium iodide (PI) using the Dead Cell Apoptosis Kit from Thermo Fisher. Cells were resuspended in Annexin V-binding buffer provided with the kit supplemented with 1 μg/mL of PI and with Annexin V-FITC 7 mM for 15 min (100 μL total volume). Thereafter, 400 μL of Annexin-binding buffer was added to each sample and the fluorescence of cells was measured through flow cytometry (for Annexin V-FITC: excitation 488 nm, emission 525 ± 40 nm; for PI: excitation 488 nm, emission 610 ± 20 nm). Data were analyzed with the software CytExpert (Beckman Coulter).

### Mitochondrial Morphology and ΔΨ_m_ Analysis

SH-SY5Y cells (10,000 cells/cm^2^) were seeded in a Willco Petri dish (GWST-3512) and incubated as previously described (24 h proliferation medium, 72 h differentiation medium, 72 h of differentiation medium plus 100 μg/mL of L-PDNPs). Thereafter, cells were treated with 5 mM TBH for 40 min and then stained with 1 μM TMRM (Life Technologies) for 1 h in differentiation cell culture medium, washed twice with DPBS (Sigma), incubated with HEPES-supplemented (25 mM) phenol red-free DMEM (Thermo Fisher), and imaged with a confocal microscope (C2s system, Nikon). For the analysis of mitochondrial morphology, images of four different conditions were acquired (control, L-PDNPs, TBH, and L-PDNPs + TBH), and the roundness of mitochondria was measured with ImageJ using the following formula
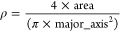
2

For ΔΨ_m_ analysis, time-lapse acquisition of cells treated as previously described was performed. Images of mitochondria stained with TMRM were acquired every 30 s for 800 s. After 120 s from the beginning of the acquisition, oligomycin 6 μM (Sigma) was added. The variation caused by oligomycin on the ΔΨ_m_ level was evaluated in different experimental conditions (control, TBH, and L-PDNPs + TBH) and expressed as % with respect to the initial value.

### Effects on Neuronal Differentiation

For the analysis of L-PDNPs effect on neuronal differentiation, cells were seeded in 3 cm diameter Petri dishes (Corning) at low cellular density (500 cells/cm^2^), incubated 24 h with proliferation medium and then incubated for 72 h with differentiation medium containing 100 μg/mL of L-PDNPs (control experiments were performed in same conditions but without nanoparticles). After 72 h, cells were fixed as previously described with PFA 4% and an immunostaining procedure was carried out to mark tubulin β-III: cells were permeabilized for 20 min with Triton X-100 (0.1% in DPBS, Sigma) and then incubated for 40 min with a blocking solution of goat serum at 10%. Afterward, cells were incubated with goat serum 10% containing 0.3 μg/mL anti-tubulin β-III antibody produced in rabbit (Sigma) for 3 h and then washed three times with goat serum 10% in DPBS (Sigma) and incubated again for 40 min in goat serum 10% containing 10 μg/mL of F(ab′)2-goat anti-Rabbit IgG (H + L) Alexa Fluor 488 conjugate (Invitrogen), 2.5 μg/mL of TRITC-phalloidin (Sigma), and 5 μg/mL of Hoechst (Invitrogen). Afterward, cells were washed three times with DPBS (Sigma) and imaged through fluorescence microscopy (Eclipse Ti, Nikon) with a 10× objective. The length of neurites was measured and compared among the different experimental conditions using ImageJ. The expression of tubulin β-III was analyzed with NIS elements software: a fluorescence threshold was set, and cells immunostained against tubulin β-III were classified in tubulin β-III-positive (fluorescence levels above threshold) and tubulin β-III-negative (fluorescence levels below threshold).

### L-PDNP NIR Photothermal Conversion Ability

The absorbance of L-PDNP dispersions in Milli-Q water at different concentrations (30, 62.75, 125, and 250 μg/mL) in the 700–1000 nm window was measured using a PerkinElmer UV/vis spectrophotometer (Lambda 45). The photothermal conversion ability of L-PDNPs was measured using an EL-USB-1 temperature data logger thermocouple: increasing concentrations of L-PDNPs in 1 mL of Milli-Q water (0.1, 0.25, 0.5, 1, and 5 mg/mL) were placed in a 3 cm Petri dish (Corning) and irradiated with a RLTMDL-808-500 NIR laser (λ = 808 nm), with a spot size of 2.5 mm and a laser power of 532 mW for 60 s. For the temperature measurement, the thermocouple was placed in proximity to the laser spot. For the evaluation of the photothermal conversion effect of L-PDNPs on SH-SY5Y, 10,000 cells/cm^2^ were seeded in a Willco Petri dish (GWST-3512) with glass bottom and incubated as previously described (24 h proliferation medium, 72 h of differentiation medium, and other 72 h of differentiation medium plus 100 μg/mL of L-PDNPs; controls cultures without addition of nanoparticles were performed as well). After the incubation, cells were stained with ER Thermo Yellow (300 nM for 30 min at 37 °C), a fluorescent temperature-sensitive dye. Cells were then irradiated with a RLTMDL-808-500 NIR laser (λ = 808 nm) with a 2.5 mm laser spot diameter and imaged through laser scanning confocal microscopy time-lapse imaging (C2s system, Nikon) using a 20× objective and acquiring images every 15 s for a total span of 660 s (images were acquired from three different fields). Cultures underwent stimulation with different laser powers during the imaging (0 mW from *t* = 0 s to *t* = 60 s, 0.148 mW from *t* = 60 s to *t* = 120 s, 0.425 mW from *t* = 120 s to *t* = 180 s, 1 mW from *t* = 180 s to *t* = 240 s, 69.5 mW from *t* = 240 s to *t* = 300 s, 178 mW from *t* = 300 s to *t* = 360 s, 286.5 mW from *t* = 360 s to *t* = 420 s, 395 mW from *t* = 420 s to *t* = 480 s, 532.5 from *t* = 480 s to *t* = 540 s, and 0 mW from *t* = 540 s to *t* = 660 s) and the relative reduction in fluorescence caused by heating was measured and presented as *F*/*F*_0_. The increment in temperature was derived by converting every 2.8% of fluorescence decrement into a 1 °C temperature increment, calculated from the work of Arai *et al.*(48) and considering 25 °C as the starting temperature of the cells.

### Theoretical Model for Temperature Increment Mediated by Nanoparticles and NIR Radiation

In light of the experimental setup schematized in Figure S14a, we initially considered an axisymmetric approximation also involving the liquid domain (phenol red-free DMEM high glucose with 1% FBS and 25 mM HEPES, approximated as water). However, based on preliminary estimates, we soon opted for a simpler formulation. Indeed, with reference to Figure S14b, heat generation was expected to occur on the thin glass substrate (height *h* = 0.2 mm, radius *r*_∞_ = 6 mm), in particular thanks to the L-PDNPs reached by the irradiation spot (radius *r*_spot_ = 1.25 mm). Moreover, a thermal exchange between glass and water was expected to be larger than that one between glass and air, based on relevant thermal exchange coefficients: γ_w_ ≅ 10^2^ and γ_a_ ≅ 10^1^ W m^–2^ K^–1^ for water and air, respectively.^49^ Furthermore, heat capacity was smaller for the glass substrate compared to water, as it can be estimated by considering, in particular, glass density ρ ≅ 2.5 10^3^ kg m^–3^ and specific heat *c* ≅ 8.4 10^2^ J kg^–1^ K^–1^^50^ (besides differences in volume, see Figure S14b). We thus assumed temperature variations in the liquid to be negligible, and we focused on heat transfer in the glass substrate by considering the 1D radial domain sketched in Figure S14c (where height *h* is graphically kept for ease of rendering: in the following derivation, it only indirectly appears, associated with volume).

Denoting by *T* = *T*(*r*, *t*) the unknown temperature, as a function of space (*r*) and time (*t*) variables, we introduced the following partial differential equation by imposing the energy balance to an elementary control volume
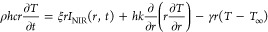
3where *T*_∞_ = 298 K denotes the (constant) room temperature outside glass, γ ≔ γ_w_ + γ_a_, and *k* ≅ 1.1 W m^–1^ K^–1^ represents glass thermal conductivity.^51^ With reference to Figure S14c, −γ*r*(*T* – *T*_∞_) models thermal exchange (*te*), while  accounts for conductive heat flux (*q*), based on the classical Fourier’s law *q* = −*k*∇*T*. Moreover, *I*_NIR_ denotes the nominal NIR intensity, namely, zero for *r*_spot_ < *r* ≤ *r*_∞_ and *P*_NIR_/(π*r*^2^_spot_) for 0 ≤ *r* ≤ *r*_spot_, where *P*_NIR_ = *P*_NIR_(*t*) is the given time law for the input NIR power. Finally, parameter 0 < ξ < 1 accounts for the NIR power fraction that actually acts as a heat source. Let us remark that, although heat was generated on the upper surface of the glass substrate onto which cells with associated L-PDNPs adhered, we introduced a volumetric source consistently with 1D approximation. Ideally, the considered fraction can be represented as ξ ≔ =ξ_NPdensity_ξ_NPabs_, where ξ_NPdensity_ is a “covering/occupancy” fraction based on particles density in the irradiated region, and fraction ξ_NPabs_ corresponds to the effective absorption coefficient of the particles. For our experiments, we derived ξ_NPdensity_ ≅ 0.17 based on standard image processing (binarization, see Figure S15a,b) implemented in Matlab (The Mathworks). Moreover, considering the measured transmittance coefficient (around 0.75, see right below), we estimated ξ_NPabs_ < 0.25 (the latter figure including both adsorption and reflection/backscattering). Considering the model scope and objective, we kept ξ as a calibration parameter (see below). The aforementioned transmittance values of L-PDNPs associated with SH-SY5Y was derived by measuring the absorbance, with a PerkinElmer UV/vis spectrophotometer (Lambda 45), of both plain and L-PDNPs-treated (100 μg/mL) SH-SY5Y cells seeded in a Willco Petri dish (GWST-3512), fixed with glutaraldehyde, and dehydrated as previously described. In order to integrate eq 3, we introduced a nondimensional formulation, in particular, for obtaining *T̅* ≔ (*T* – *T*_∞_)/*T*_∞_. For the purpose, we chose  ≔ *r*_spot_ as reference length and *t̅* ≔ *t*/*t*_∞_ as reference time and we adopted *r̅* ≔  and *t̅* ≔ *t*/*t*_∞_ as independent variables, thus recasting eq 3 as follows
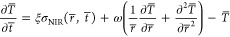
4where ω ≔  accounts for the relative importance of conduction and thermal exchange and
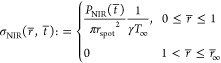
5with *r̅*_∞_ ≔ . In order to close the differential problem, we considered a uniform initial condition *T̅*(*r̅*, *t̅* = 0) = 0, and we imposed an unperturbed far-field condition *T̅*(*r̅* = *r̅*_∞_,*t̅*) = 0 as a working assumption (to be checked upon integration). Finally, we imposed  by symmetry. The resulting differential problem stemming from eqs 4 and 5 and the aforementioned boundary and initial conditions are explicitly written in Results and Discussion (eq 1), for ease of presentation.

The derived nondimensional problem was numerically integrated by adopting the method of lines.^52^ In particular, we used second-order-accurate-centered finite differences for the spatial derivatives, and we directly embedded the boundary conditions through second-order-accurate one-sided differences. Moreover, the resulting ordinary differential equation was integrated in time by using a standard fourth–fifth order explicit Runge–Kutta scheme available through Matlab. The obtained (dimensional) numerical results are shown in Figure 8c, together with experimental data. Upon calibration (least-square error minimization with respect to said data), we derived ξ_NPabs_ ≅ 0.17, consistently with the aforementioned (upper bound) inequality. The obtained results also confirmed the working assumption of the unperturbed far field.

### Calcium Imaging

For the analysis of variation in Ca^2+^ intracellular levels during NIR irradiation, cells were seeded in a Willco Petri dish (GWST-3512) and treated as previously described (24 h proliferation medium, 72 h of differentiation medium, and other 72 h of differentiation medium plus 100 μg/mL of L-PDNPs; control cultures without nanoparticles have been used as well). After the incubation, cells were stained with Fluo-4 AM (1 μM, Invitrogen) for 30 min at 37 °C and, subsequently, samples were rinsed with DPBS (Sigma) and incubated with HEPES-supplemented (25 mM) phenol red-free DMEM (Thermo Fisher) for laser scanning confocal microscopy time-lapse imaging (C2s system, Nikon). Control cells and cells incubated with 100 μg/mL L-PDNPs were irradiated with an RLTMDL-808-500 NIR laser (λ = 808 nm) with a 2.5 mm spot diameter and 532 mW of laser power for 450 s. Images were acquired from three different fields every 15 s with a 20× objective.

### ROS Analysis Following NIR Stimulation

In order to assess the production of ROS after NIR irradiation, SH-SY5Y were seeded in Willco Petri dishes at 10,000 cells/cm^2^ and treated as previously described (24 h proliferation medium, 72 h of differentiation medium, and other 72 h of differentiation medium plus 100 μg/mL of L-PDNPs; control cultures without nanoparticles have been used as well). Then, samples were rinsed with DPBS (Sigma), incubated with HEPES-supplemented (25 mM) phenol red-free DMEM (Thermo Fisher), and irradiated with an RLTMDL-808-500 NIR laser (λ = 808 nm) with 2.5 mm spot diameter and 532 mW of laser power for 450 s. After the irradiation, cells were stained with CellROX as previously described, and the relative fluorescence of four different experimental conditions (control, L-PDNPs, NIR, and NIR + L-PDNPs) again measured through flow cytometry.

### Statistical Analysis

Statistical analysis was carried out using the software R. The normality of data distribution was tested with the Shapiro–Wilk test; normally-distributed data were analyzed with ANOVA followed by LSD *post hoc* with Bonferroni correction and expressed as average ± standard error. Non-normally distributed data were analyzed with the Kruskal–Wallis test followed by pairwise Wilcox *post hoc* test with Holm correction and expressed as median ± 95% confidence interval. Each experiment has been performed in triplicate (*n* = 3), if not differently indicated.

## Supporting Information Available

L-PDNP characterization including DLS, *Z*-potential, and BET measurements; TGA analysis of mPEG-DSPE; PicoGreen and LIVE/DEAD assays on SH-SY5Y cells treated with different concentrations of L-PDNPs; images of SH-SY5Y treated with different concentrations of L-PDNPs and stained with LIVE/DEAD reagents; 3D confocal rendering of SH-SY5Y internalizing DiO-L-PDNPs; histograms showing fluorescence levels of SH-SY5Y treated with DiO-L-PDNPs at various time points; confocal images showing the intracellular co-localization of DiO-L-PDNPs with lysosomes and mitochondria; confocal images of bEnd.3 cells and analysis of the passage of FITC-dextran 70 kDa across an *in vitro* BBB model; 3D confocal rendering of bEnd.3 cells treated with DiO-L-PDNPs for 72 h; flow cytometry plot showing Annexin V-FITC/PI analysis; absorbance spectra of L-PDNPs and photothermal conversion properties of dispersions of L-PDNPs at different concentrations; analysis of the production of ROS in SH-SY5Y cells after NIR stimulation; schematic model of the NIR stimulation experiment; and SEM images showing L-PDNP interactions with SH-SY5Y cells (PDF)

## References

[ref1] Martinelli C.; Pucci C.; Battaglini M.; Marino A.; Ciofani G. Antioxidants and Nanotechnology: Promises and Limits of Potentially Disruptive Approaches in the Treatment of Central Nervous System Diseases. Adv. Healthcare Mater. 2020, 9, 1901589 10.1002/adhm.201901589. 31854132

[ref2] Dias V.; Junn E.; Mouradian M. M. The Role of Oxidative Stress in Parkinson’s Disease. J. Parkinson’s Dis. 2013, 3, 461–491. 10.3233/jpd-130230. 24252804 PMC4135313

[ref3] Wojsiat J.; Zoltowska K. M.; Laskowska-Kaszub K.; Wojda U. Oxidant/Antioxidant Imbalance in Alzheimer’s Disease: Therapeutic and Diagnostic Prospects. Oxid. Med. Cell. Longevity 2018, 2018, 6435861 10.1155/2018/6435861. PMC5831771 29636850

[ref4] Gil-Mohapel J.; Brocardo P.; Christie B. The Role of Oxidative Stress in Huntington’s Disease: Are Antioxidants Good Therapeutic Candidates?. Curr. Drug Targets 2014, 15, 454–468. 10.2174/1389450115666140115113734. 24428525

[ref5] Olmez I.; Ozyurt H. Reactive Oxygen Species and Ischemic Cerebrovascular Disease. Neurochem. Int. 2012, 60, 208–212. 10.1016/j.neuint.2011.11.009. 22122807

[ref6] Ohl K.; Tenbrock K.; Kipp M. Oxidative Stress in Multiple Sclerosis: Central and Peripheral Mode of Action. Exp. Neurol. 2016, 277, 58–67. 10.1016/j.expneurol.2015.11.010. 26626971 PMC7094520

[ref7] Pizzino G.; Irrera N.; Cucinotta M.; Pallio G.; Mannino F.; Arcoraci V.; Squadrito F.; Altavilla D.; Bitto A. Oxidative Stress: Harms and Benefits for Human Health. Oxid. Med. Cell. Longevity 2017, 2017, 8416763 10.1155/2017/8416763. PMC5551541 28819546

[ref8] Bélanger M.; Allaman I.; Magistretti P. J. Brain Energy Metabolism: Focus on Astrocyte-Neuron Metabolic Cooperation. Cell Metab. 2011, 14, 724–738. 10.1016/j.cmet.2011.08.016. 22152301

[ref9] Nissanka N.; Moraes C. T. Mitochondrial DNA Damage and Reactive Oxygen Species in Neurodegenerative Disease. FEBS Lett. 2018, 592, 728–742. 10.1002/1873-3468.12956. 29281123 PMC6942696

[ref10] Celardo I.; Pedersen J. Z.; Traversa E.; Ghibelli L. Pharmacological Potential of Cerium Oxide Nanoparticles. Nanoscale 2011, 3, 1411–1420. 10.1039/c0nr00875c. 21369578

[ref11] Hamasaki T.; Kashiwagi T.; Imada T.; Nakamichi N.; Aramaki S.; Toh K.; Morisawa S.; Shimakoshi H.; Hisaeda Y.; Shirahata S. Kinetic Analysis of Superoxide Anion Radical-Scavenging and Hydroxyl Radical-Scavenging Activities of Platinum Nanoparticles. Langmuir 2008, 24, 7354–7364. 10.1021/la704046f. 18553993

[ref12] Singh N.; Savanur M. A.; Srivastava S.; D’Silva P.; Mugesh G. A Redox Modulatory Mn(3) O(4) Nanozyme with Multi-Enzyme Activity Provides Efficient Cytoprotection to Human Cells in a Parkinson’s Disease Model. Angew. Chem., Int. Ed. Engl. 2017, 56, 14267–14271. 10.1002/anie.201708573. 28922532

[ref13] Singh N.; Savanur M. A.; Srivastava S.; D’Silva P.; Mugesh G. A Manganese Oxide Nanozyme Prevents the Oxidative Damage of Biomolecules without Affecting the Endogenous Antioxidant System. Nanoscale 2019, 11, 3855–3863. 10.1039/c8nr09397k. 30758009

[ref14] Marino A.; Tonda-Turo C.; De Pasquale D.; Ruini F.; Genchi G.; Nitti S.; Cappello V.; Gemmi M.; Mattoli V.; Ciardelli G.; Ciofani G. Gelatin/Nanoceria Nanocomposite Fibers as Antioxidant Scaffolds for Neuronal Regeneration. Biochim. Biophys. Acta, Gen. Subj. 2017, 1861, 386–395. 10.1016/j.bbagen.2016.11.022. 27864151

[ref15] Ciofani G.; Genchi G. G.; Liakos I.; Cappello V.; Gemmi M.; Athanassiou A.; Mazzolai B.; Mattoli V. Effects of Cerium Oxide Nanoparticles on PC12 Neuronal-Like Cells: Proliferation, Differentiation, and Dopamine Secretion. Pharm. Res. 2013, 30, 2133–2145. 10.1007/s11095-013-1071-y. 23661146

[ref16] Ciofani G.; Genchi G. G.; Mazzolai B.; Mattoli V. Transcriptional Profile of Genes Involved in Oxidative Stress and Antioxidant Defense in PC12 Cells Following Treatment with Cerium Oxide Nanoparticles. Biochim. Biophys. Acta, Gen. Subj. 2014, 1840, 495–506. 10.1016/j.bbagen.2013.10.009. 24135455

[ref17] Genchi G. G.; Degl’Innocenti A.; Salgarella A. R.; Pezzini I.; Marino A.; Menciassi A.; Piccirillo S.; Balsamo M.; Ciofani G. Modulation of Gene Expression in Rat Muscle Cells Following Treatment with Nanoceria in Different Gravity Regimes. Nanomedicine 2018, 13, 2821–2833. 10.2217/nnm-2018-0316. 30334476

[ref18] Pezzini I.; Marino A.; Del Turco S.; Nesti C.; Doccini S.; Cappello V.; Gemmi M.; Parlanti P.; Santorelli F. M.; Mattoli V.; Ciofani G. Cerium Oxide Nanoparticles: The Regenerative Redox Machine in Bioenergetic Imbalance. Nanomedicine 2017, 12, 403–416. 10.2217/nnm-2016-0342. 28000542

[ref19] Battaglini M.; Tapeinos C.; Cavaliere I.; Marino A.; Ancona A.; Garino N.; Cauda V.; Palazon F.; Debellis D.; Ciofani G. Design, Fabrication, and In Vitro Evaluation of Nanoceria-Loaded Nanostructured Lipid Carriers for the Treatment of Neurological Diseases. ACS Biomater. Sci. Eng. 2019, 5, 670–682. 10.1021/acsbiomaterials.8b01033. 33405830

[ref20] Rocca A.; Moscato S.; Ronca F.; Nitti S.; Mattoli V.; Giorgi M.; Ciofani G. Pilot in Vivo Investigation of Cerium Oxide Nanoparticles as a Novel Anti-Obesity Pharmaceutical Formulation. Nanomedicine 2015, 11, 1725–1734. 10.1016/j.nano.2015.05.001. 26003299

[ref21] Yokel R. A.; Au T. C.; MacPhail R.; Hardas S. S.; Butterfield D. A.; Sultana R.; Goodman M.; Tseng M. T.; Dan M.; Haghnazar H.; Unrine J. M.; Graham U. M.; Wu P.; Grulke E. A. Distribution, Elimination, and Biopersistence to 90 Days of a Systemically Introduced 30 Nm Ceria-Engineered Nanomaterial in Rats. Toxicol. Sci. 2012, 127, 256–268. 10.1093/toxsci/kfs067. 22367688

[ref22] Liu Y.; Ai K.; Lu L. Polydopamine and Its Derivative Materials: Synthesis and Promising Applications in Energy, Environmental, and Biomedical Fields. Chem. Rev. 2014, 114, 5057–5115. 10.1021/cr400407a. 24517847

[ref23] Bao X.; Zhao J.; Sun J.; Hu M.; Yang X. Polydopamine Nanoparticles as Efficient Scavengers for Reactive Oxygen Species in Periodontal Disease. ACS Nano 2018, 12, 8882–8892. 10.1021/acsnano.8b04022. 30028940

[ref24] Srivastava A. K.; Roy Choudhury S.; Karmakar S. Melatonin/Polydopamine Nanostructures for Collective Neuroprotection-Based Parkinson’s Disease Therapy. Biomater. Sci. 2020, 8, 1345 10.1039/c9bm01602c. 31912833

[ref25] Zhao H.; Zeng Z.; Liu L.; Chen J.; Zhou H.; Huang L.; Huang J.; Xu H.; Xu Y.; Chen Z.; Wu Y.; Guo W.; Wang J. H.; Wang J.; Liu Z. Polydopamine Nanoparticles for the Treatment of Acute Inflammation-Induced Injury. Nanoscale 2018, 10, 6981–6991. 10.1039/c8nr00838h. 29610822

[ref26] Sardoiwala M. N.; Srivastava A. K.; Kaundal B.; Karmakar S.; Choudhury S. R. Recuperative Effect of Metformin Loaded Polydopamine Nanoformulation Promoting EZH2 Mediated Proteasomal Degradation of Phospho-α-Synuclein in Parkinson’s Disease Model. Nanomedicine 2020, 24, 102088 10.1016/j.nano.2019.102088. 31476446

[ref27] Li M.; Sun X.; Zhang N.; Wang W.; Yang Y.; Jia H.; Liu W. NIR-Activated Polydopamine-Coated Carrier-Free “Nanobomb” for In Situ On-Demand Drug Release. Adv. Sci. 2018, 5, 1800155 10.1002/advs.201800155. PMC6051140 30027047

[ref28] Liu J.-S.; Peng S.-J.; Li G.-F.; Zhao Y.-X.; Meng X.-Y.; Yu X.-R.; Li Z.-H.; Chen J.-M. Polydopamine Nanoparticles for Deep Brain Ablation via Near-Infrared Irradiation. ACS Biomater. Sci. Eng. 2020, 6, 664–672. 10.1021/acsbiomaterials.9b01097. 33463219

[ref29] Trouillas P.; Calliste C.-A.; Allais D.-P.; Simon A.; Marfak A.; Delage C.; Duroux J.-L. Antioxidant, Anti-Inflammatory and Antiproliferative Properties of Sixteen Water Plant Extracts Used in the Limousin Countryside as Herbal Teas. Food Chem. 2003, 80, 399–407. 10.1016/s0308-8146(02)00282-0.

[ref30] Marino A.; Camponovo A.; Degl’Innocenti A.; Bartolucci M.; Tapeinos C.; Martinelli C.; De Pasquale D.; Santoro F.; Mollo V.; Arai S.; Suzuki M.; Harada Y.; Petretto A.; Ciofani G. Multifunctional Temozolomide-Loaded Lipid Superparamagnetic Nanovectors: Dual Targeting and Disintegration of Glioblastoma Spheroids by Synergic Chemotherapy and Hyperthermia Treatment. Nanoscale 2019, 11, 21227–21248. 10.1039/c9nr07976a. 31663592 PMC6867905

[ref31] Martinelli C.; Battaglini M.; Pucci C.; Gioi S.; Caracci C.; Macaluso G.; Doccini S.; Santorelli F. M.; Ciofani G. Development of Nanostructured Lipid Carriers for the Delivery of Idebenone in Autosomal Recessive Spastic Ataxia of Charlevoix-Saguenay. ACS Omega 2020, 5, 12451–12466. 10.1021/acsomega.0c01282. 32548430 PMC7271403

[ref32] Tapeinos C.; Battaglini M.; Ciofani G. Advances in the Design of Solid Lipid Nanoparticles and Nanostructured Lipid Carriers for Targeting Brain Diseases. J. Controlled Release 2017, 264, 306–332. 10.1016/j.jconrel.2017.08.033. PMC6701993 28844756

[ref33] Wu S.; Zhou F.; Zhang Z.; Xing D. Mitochondrial Oxidative Stress Causes Mitochondrial Fragmentation via Differential Modulation of Mitochondrial Fission-Fusion Proteins. FEBS J. 2011, 278, 941–954. 10.1111/j.1742-4658.2011.08010.x. 21232014

[ref34] Fan X.; Hussien R.; Brooks G. A. H2O2-Induced Mitochondrial Fragmentation in C2C12 Myocytes. Free Radical Biol. Med. 2010, 49, 1646–1654. 10.1016/j.freeradbiomed.2010.08.024. 20801212 PMC2970628

[ref35] Jendrach M.; Mai S.; Pohl S.; Vöth M.; Bereiter-Hahn J. Short- and Long-Term Alterations of Mitochondrial Morphology, Dynamics and MtDNA after Transient Oxidative Stress. Mitochondrion 2008, 8, 293–304. 10.1016/j.mito.2008.06.001. 18602028

[ref36] Barsoum M. J.; Yuan H.; Gerencser A. A.; Liot G.; Kushnareva Y.; Gräber S.; Kovacs I.; Lee W. D.; Waggoner J.; Cui J.; White A. D.; Bossy B.; Martinou J.-C.; Youle R. J.; Lipton S. A.; Ellisman M. H.; Perkins G. A.; Bossy-Wetzel E. Nitric Oxide-Induced Mitochondrial Fission Is Regulated by Dynamin-Related GTPases in Neurons. EMBO J. 2006, 25, 3900–3911. 10.1038/sj.emboj.7601253. 16874299 PMC1553198

[ref37] Bhang S. H.; Kwon S.-H.; Lee S.; Kim G. C.; Han A. M.; Kwon Y. H. K.; Kim B.-S. Enhanced Neuronal Differentiation of Pheochromocytoma 12 Cells on Polydopamine-Modified Surface. Biochem. Biophys. Res. Commun. 2013, 430, 1294–1300. 10.1016/j.bbrc.2012.11.123. 23261471

[ref38] Kang K.; Choi I. S.; Nam Y. A Biofunctionalization Scheme for Neural Interfaces Using Polydopamine Polymer. Biomaterials 2011, 32, 6374–6380. 10.1016/j.biomaterials.2011.05.028. 21652066

[ref39] Bórquez D. A.; Urrutia P. J.; Wilson C.; van Zundert B.; Núñez M. T.; González-Billault C. Dissecting the Role of Redox Signaling in Neuronal Development. J. Neurochem. 2016, 137, 506–517. 10.1111/jnc.13581. 26875993

[ref40] Tsatmali M.; Walcott E. C.; Makarenkova H.; Crossin K. L. Reactive Oxygen Species Modulate the Differentiation of Neurons in Clonal Cortical Cultures. Mol. Cell. Neurosci. 2006, 33, 345–357. 10.1016/j.mcn.2006.08.005. 17000118 PMC1797198

[ref41] Zhang S.; Hao M.; Gao W.; Liu F.; Duan J.; Kong Y.; Liu D.; Liu H. Neuron-like Cell Differentiation of HADSCs Promoted by a Copper Sulfide Nanostructure Mediated Plasmonic Effect Driven by near-Infrared Light. Nanoscale 2020, 12, 9833–9841. 10.1039/d0nr02319a. 32342083

[ref42] Marino A.; Arai S.; Hou Y.; Degl’Innocenti A.; Cappello V.; Mazzolai B.; Chang Y.-T.; Mattoli V.; Suzuki M.; Ciofani G. Gold Nanoshell-Mediated Remote Myotube Activation. ACS Nano 2017, 11, 2494–2508. 10.1021/acsnano.6b08202. 28107625

[ref43] Marino A.; Arai S.; Hou Y.; Sinibaldi E.; Pellegrino M.; Chang Y.-T.; Mazzolai B.; Mattoli V.; Suzuki M.; Ciofani G. Piezoelectric Nanoparticle-Assisted Wireless Neuronal Stimulation. ACS Nano 2015, 9, 7678–7689. 10.1021/acsnano.5b03162. 26168074 PMC9003232

[ref44] Ciofani G.; Danti S.; D’Alessandro D.; Ricotti L.; Moscato S.; Bertoni G.; Falqui A.; Berrettini S.; Petrini M.; Mattoli V.; Menciassi A. Enhancement of Neurite Outgrowth in Neuronal-Like Cells Following Boron Nitride Nanotube-Mediated Stimulation. ACS Nano 2010, 4, 6267–6277. 10.1021/nn101985a. 20925390

[ref45] Yong J.; Needham K.; Brown W. G. A.; Nayagam B. A.; McArthur S. L.; Yu A.; Stoddart P. R. Gold-Nanorod-Assisted near-Infrared Stimulation of Primary Auditory Neurons. Adv. Healthcare Mater. 2014, 3, 1862–1868. 10.1002/adhm.201400027. 24799427

[ref46] Li M.; Guan Y.; Zhao A.; Ren J.; Qu X. Using Multifunctional Peptide Conjugated Au Nanorods for Monitoring β-Amyloid Aggregation and Chemo-Photothermal Treatment of Alzheimer’s Disease. Theranostics 2017, 7, 2996–3006. 10.7150/thno.18459. 28839459 PMC5566101

[ref47] Sudhakar S.; Santhosh P. B.; Mani E. Dual Role of Gold Nanorods: Inhibition and Dissolution of Aβ Fibrils Induced by Near IR Laser. ACS Chem. Neurosci. 2017, 8, 2325–2334. 10.1021/acschemneuro.7b00238. 28737894

[ref48] Arai S.; Lee S.-C.; Zhai D.; Suzuki M.; Chang Y. T. A Molecular Fluorescent Probe for Targeted Visualization of Temperature at the Endoplasmic Reticulum. Sci. Rep. 2014, 4, 6701 10.1038/srep06701. 25330751 PMC4204065

[ref49] Kreith F.; Black W. Z. Basic Heat Transfer; Harper and Row, 1980.

[ref50] Tipler P. A. Physics for Scientists and Engineers, 4th ed.; W. H. Freeman, 1999.

[ref51] Cremers C. J.; Fine H. A. Thermal Conductivity; Springer US, 1990.

[ref52] Griffiths D. F.; Watson G. A. W. Numerical Analysis 1995; CRC Press, 1996.

