# Supplementary material for: Polydopamine Nanoparticles as an Organic and Biodegradable Multitasking Tool for Neuroprotection and Remote Neuronal Stimulation
Source: ACS Appl Mater Interfaces. 2020 Jul 22;12(32):35782–98. doi: 10.1021/acsami.0c05497 (PMC8009471; doi:10.1021/acsami.0c05497)

Supporting Information for

Polydopamine nanoparticles as an organic and biodegradable multitasking tool for neuroprotection and remote neuronal stimulation

Matteo Battaglini[1,2,*], Attilio Marino[1], Alessio Carmignani[1], Christos Tapeinos[1], Valentina Cauda[3], Andrea Ancona[3], Nadia Garino[3], Veronica Vighetto[3], Gabriele La Rosa[4], Edoardo Sinibaldi[5,*], Gianni Ciofani[1,*]

[1]Istituto Italiano di Tecnologia, Smart Bio-Interfaces, Viale Rinaldo Piaggio 34, 56025 Pontedera, Italy
[2]Scuola Superiore Sant'Anna, The Biorobotics Institute, Viale Rinaldo Piaggio 34, 56025 Pontedera, Italy
[3]Politecnico di Torino, Department of Applied Science and Technology, Corso Duca degli Abruzzi 24, 10129 Torino, Italy
[4]Istituto Italiano di Tecnologia, Nanochemistry, Via Morego 30, 16163 Genova, Italy
[5]Istituto Italiano di Tecnologia, Bioinspired Soft Robotics, Viale Rinaldo Piaggio 34, 56025 Pontedera, Italy

Corresponding Authors

*matteo.battaglini@iit.it; *edoardosinibaldi@iit.it; *gianni.ciofani@iit.it

Figure S1. TGA of mPEG-DSPE, showing the weight loss induced by heating from 30°C to 600°C; derivative plot is shown in red.

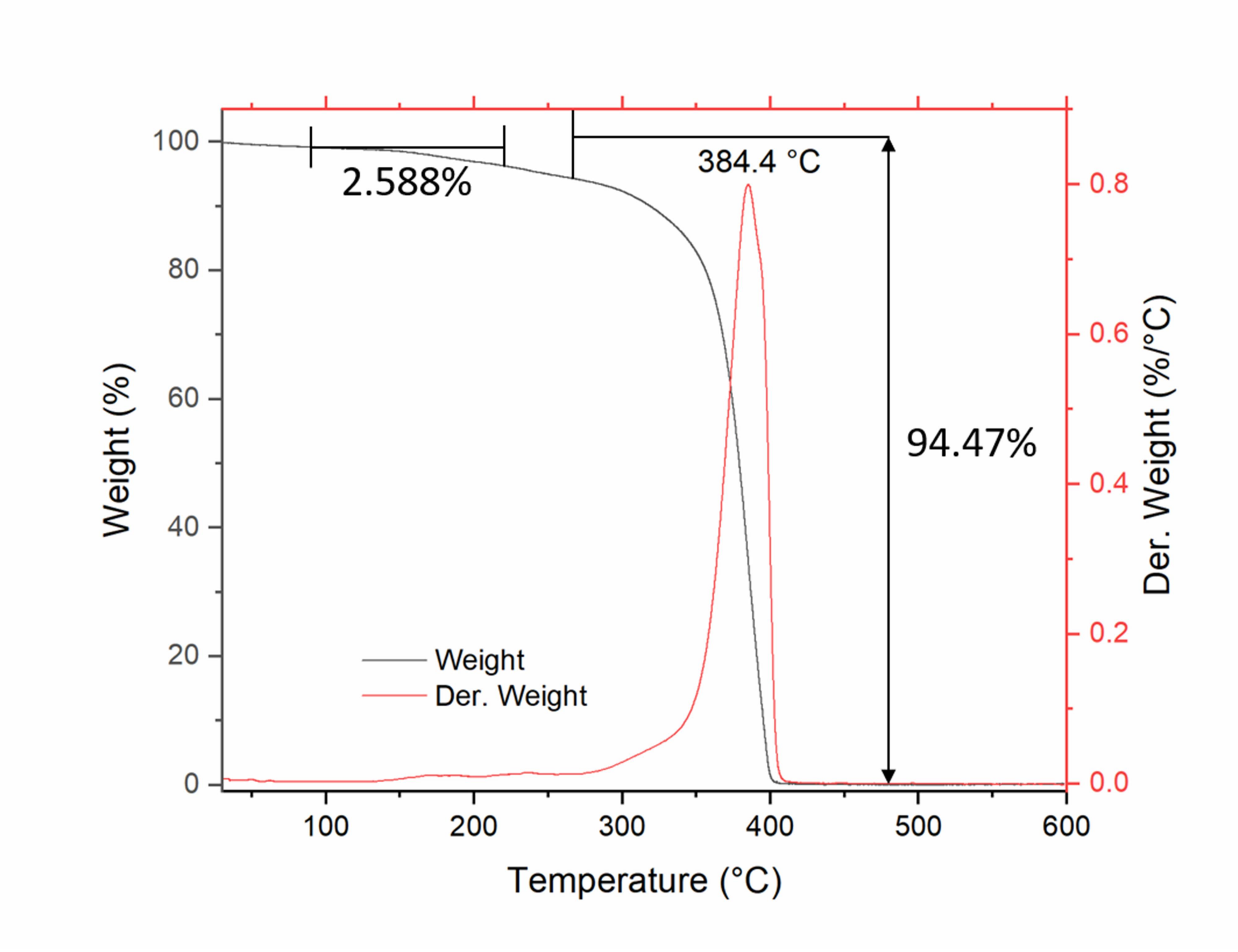

Figure S2. a) Hydrodynamic diameter distribution of L-PDNPs. b) Z-potential analysis of L-PDNPs. c) Isotherm curves generated during the adsorption and desorption of nitrogen from L-PDNPs. d) Pore size distribution in L-PDNPs.

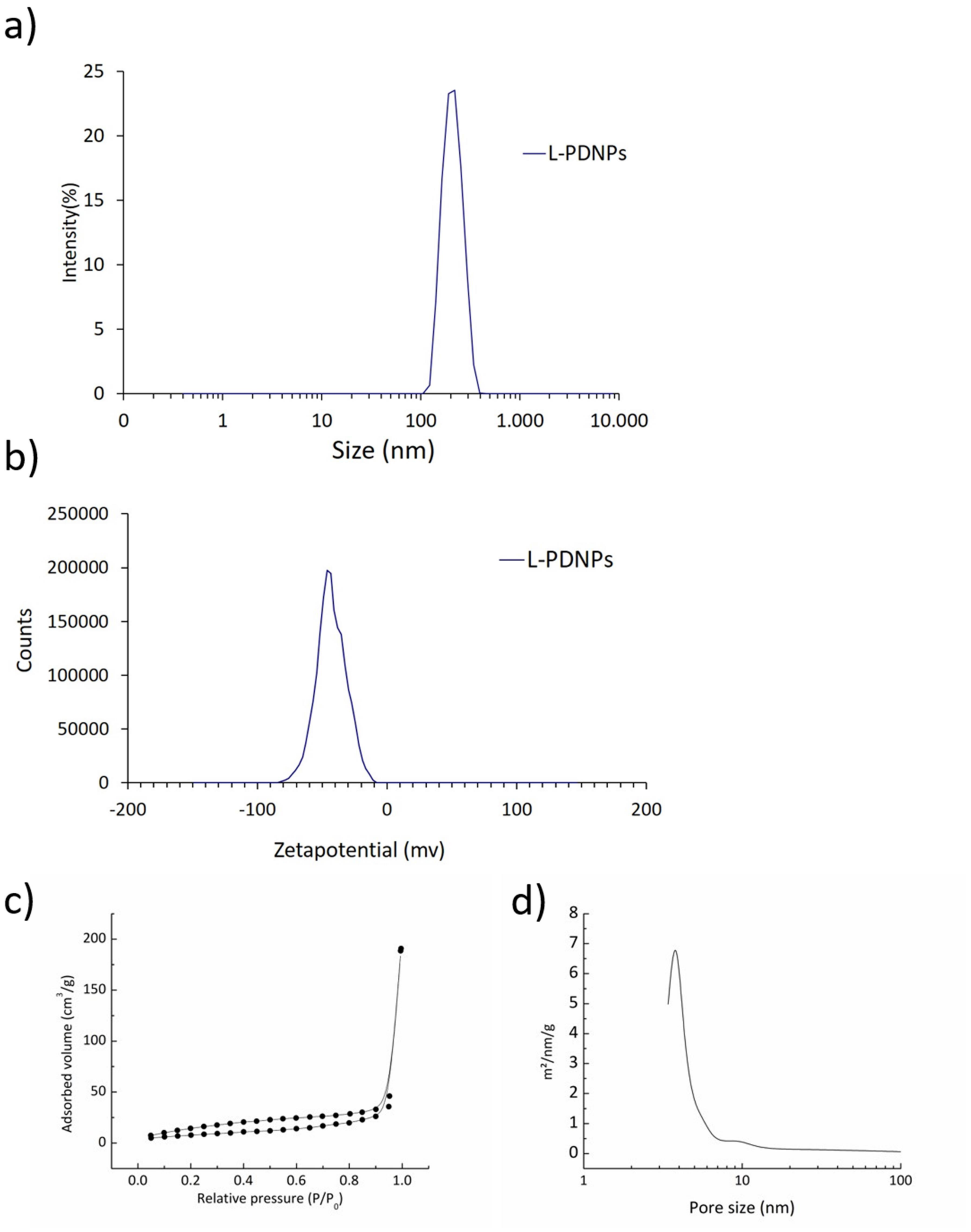

Figure S3. a) PicoGreen assay on differentiated SH-SY5Y cells incubated with different concentrations of L-PDNPs (0, 31.25, 62.5, 125 and 250 µg/ml) for 24 h (red columns) and 72 h (green columns). No statistically significant differences were observed among samples at the same time points ($n = 3$, $p > 0.05$). b) LIVE/DEAD assay on cultures treated with the same concentrations of L-PDNPs for 72 h; once again, no statistically significant differences were observed among all the samples ($n = 3$, $p > 0.05$).

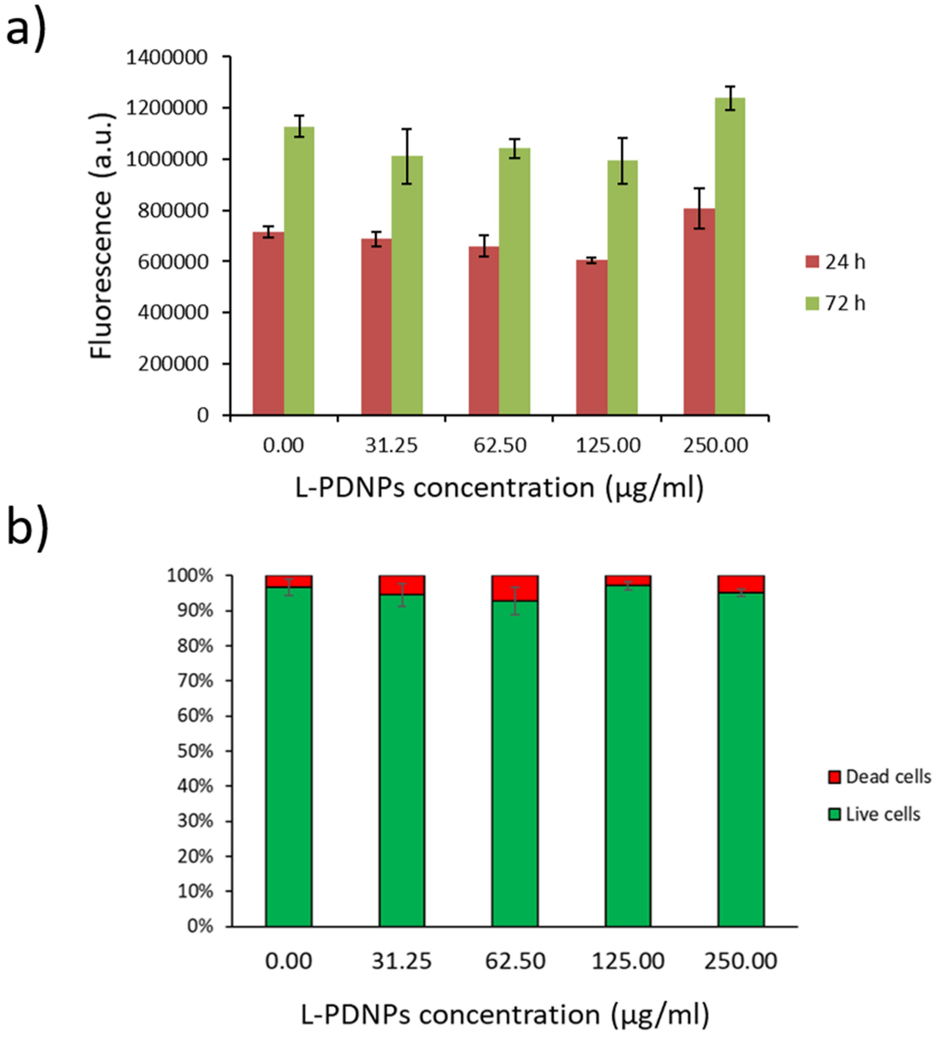

Figure S4. Representative fluorescence images of the LIVE/DEAD analysis on differentiated SH-SY5Y cells treated with increasing concentrations of L-PDNPs (0, 32.25, 67.50, 125 and 250 µg/ml) for 72 h. In green live cells stained with calcein, in red dead cells stained with ethidium homodimer, in blue nuclei stained with Hoechst.

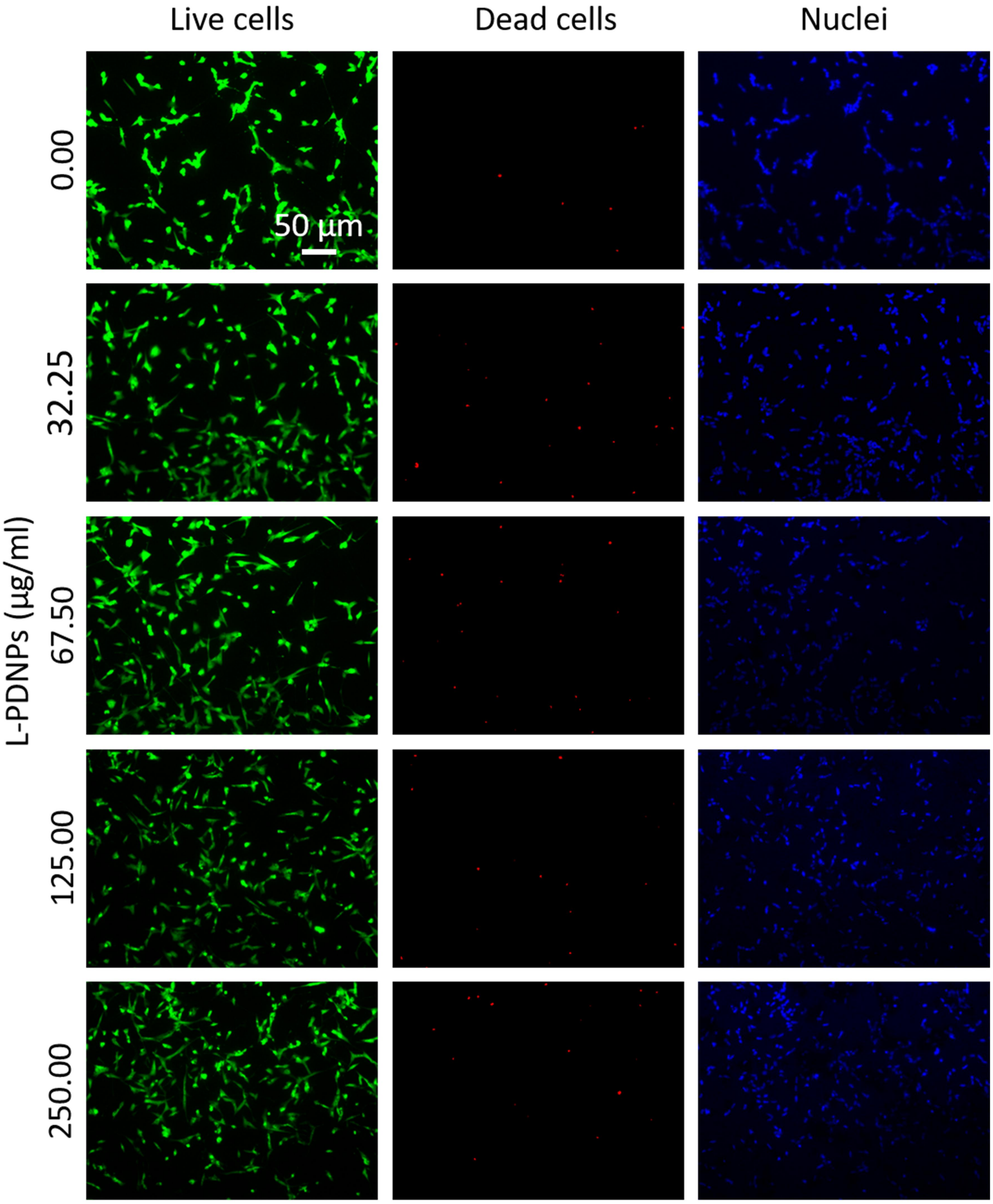

Figure S5. 3D confocal rendering of differentiated SH-SY5Y treated with DiO-L-PDNPs for increasing times (30 min, 4 h, 24 h, 72 h). In red F-actin, in green DiO-L-PDNPs, in blue nuclei.

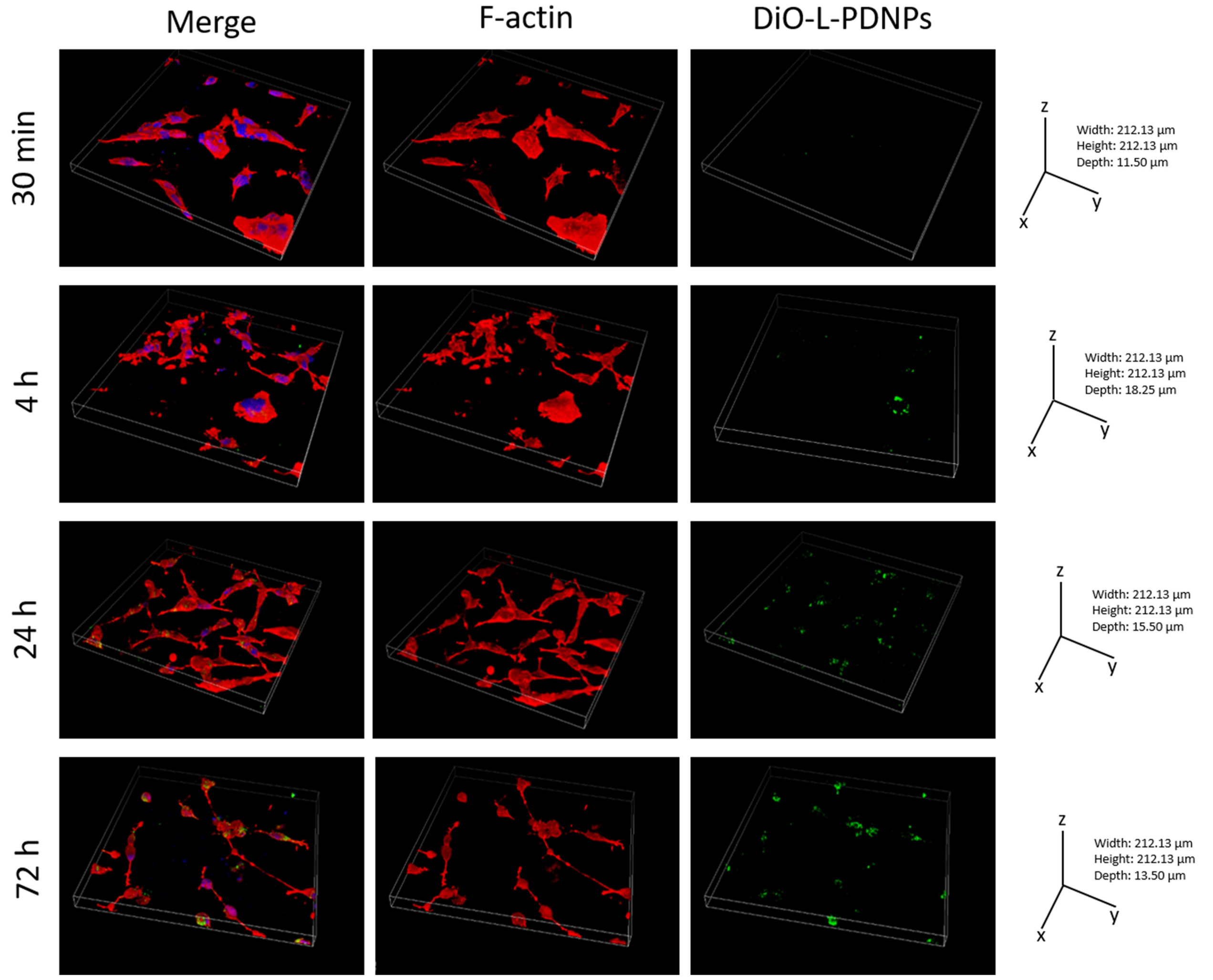

Figure S6. Representative flow cytometry data of differentiated SH-SY5Y treated with DiO-L-PDNPs for increasing times (30 min, 4 h, 24 h and 72 h). In gray fluorescence-negative cells, in green fluorescence-positive cells.

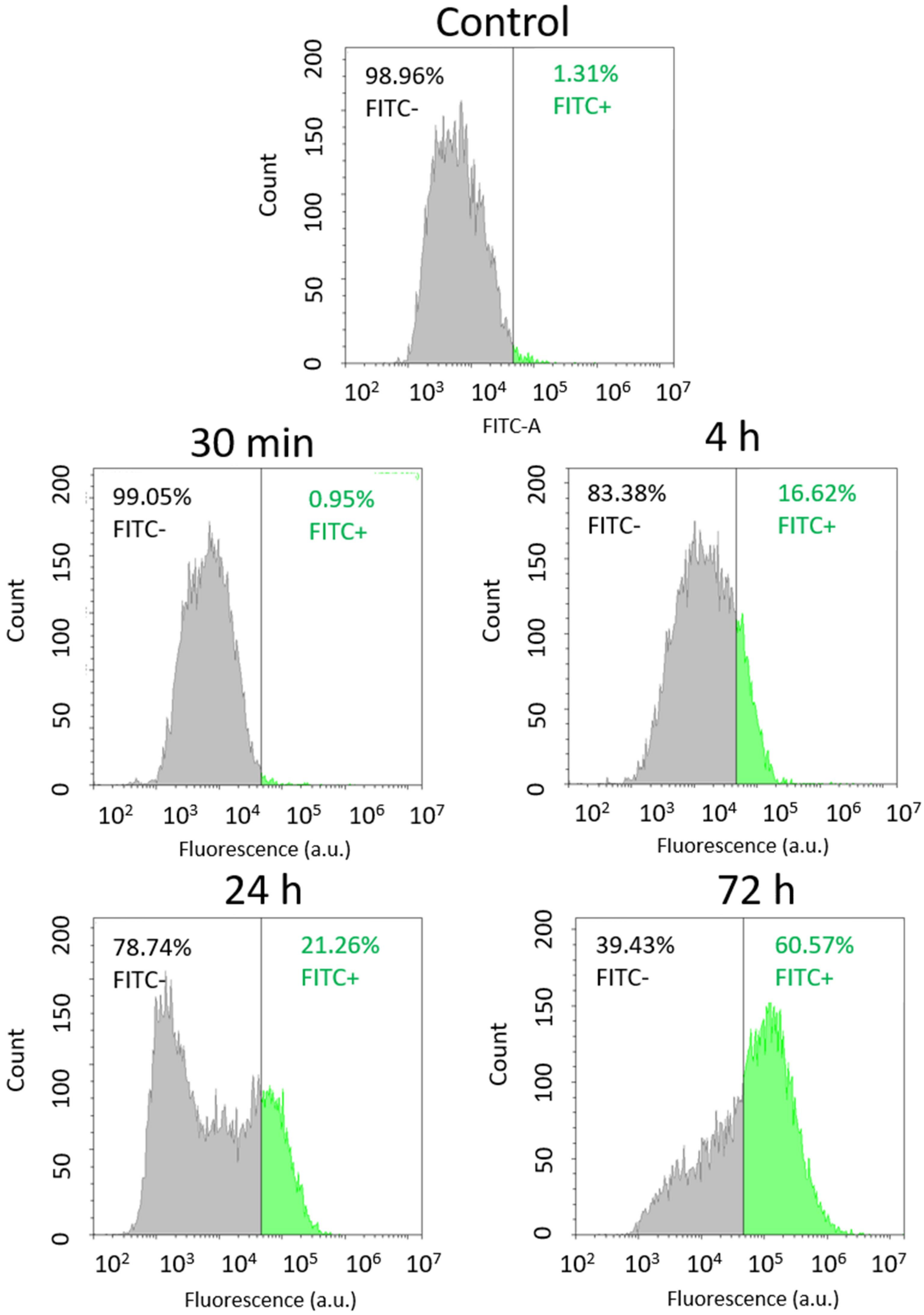

Figure S7. Analysis of the intracellular fate of DiO-L-PDNPs. a) Representative confocal images of differentiated SH-SY5Y cells incubated with DiO-L-PDNPs for increasing times (30 min, 4 h, 24 h, 72h; in green DiO-L-PDNPs, in red lysosomes, in blue nuclei). b) Pearson correlation coefficient calculated between DiO-L-PDNPs and lysosomes fluorescence at different time points (30 min is not reported due to the absence of internalized DiO-L-PDNPs at this time point; *n* = 3).

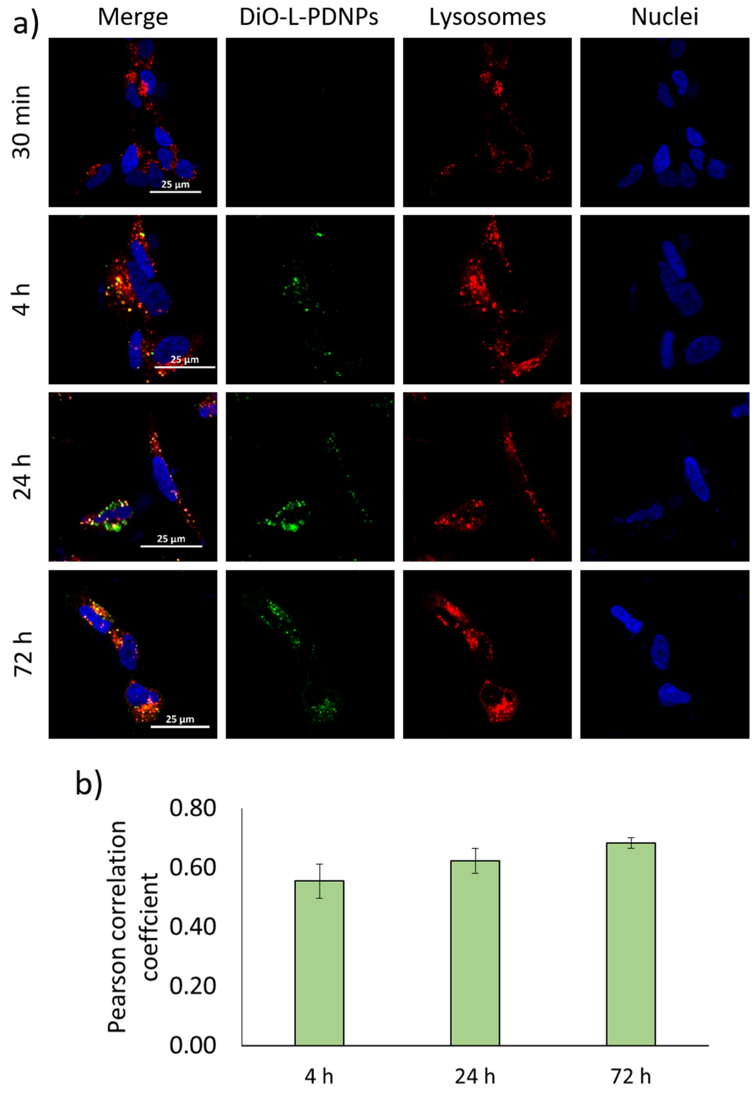

Figure S8. Analysis of the intracellular fate of DiO-L-PDNPs. a) Representative confocal images of differentiated SH-SY5Y cells incubated with DiO-L-PDNPs for increasing times (30 min, 4 h, 24 h, 72h; in green DiO-L-PDNPs, in red mitochondria, in blue nuclei). b) Pearson correlation coefficient calculated between DiO-L-PDNPs and mitochondria fluorescence at different time points (30 min is not reported due to the absence of internalized DiO-L-PDNPs at this time point; *n* = 3).

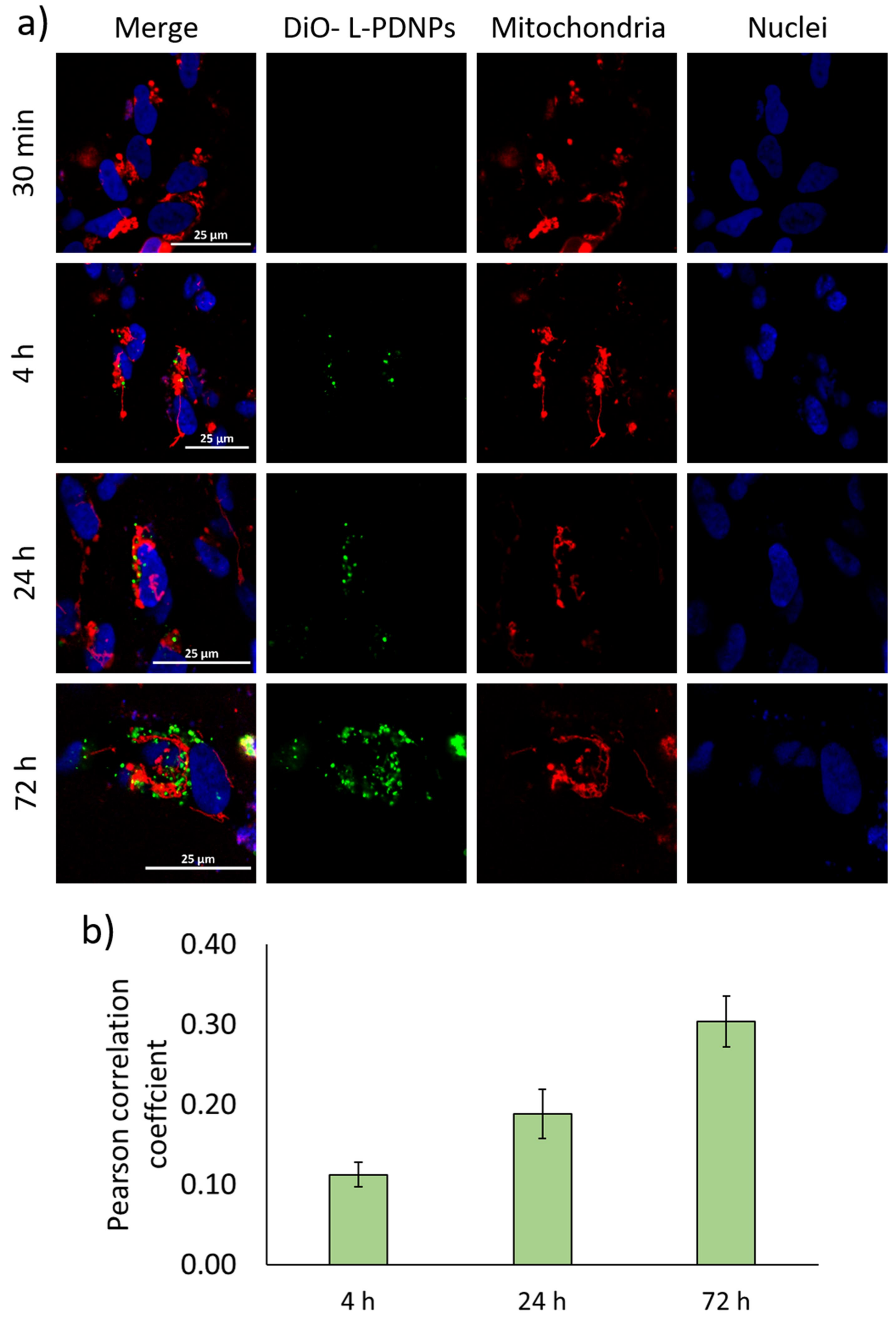

Figure S9. Characterization of the BBB *in vitro* model. a) Immunostaining showing the localization of ZO-1 in the cell-cell contact regions, suggesting the formation of tight junctions (in red F-actin, in green ZO-1, in blue nuclei). b) Analysis of the passage of fluorescent FITC-dextran 70 kDa through a transwell porous insert seeded (blue) or not (orange) with bEnd.3 cells at different time points (24 and 72 h; $n = 3$).

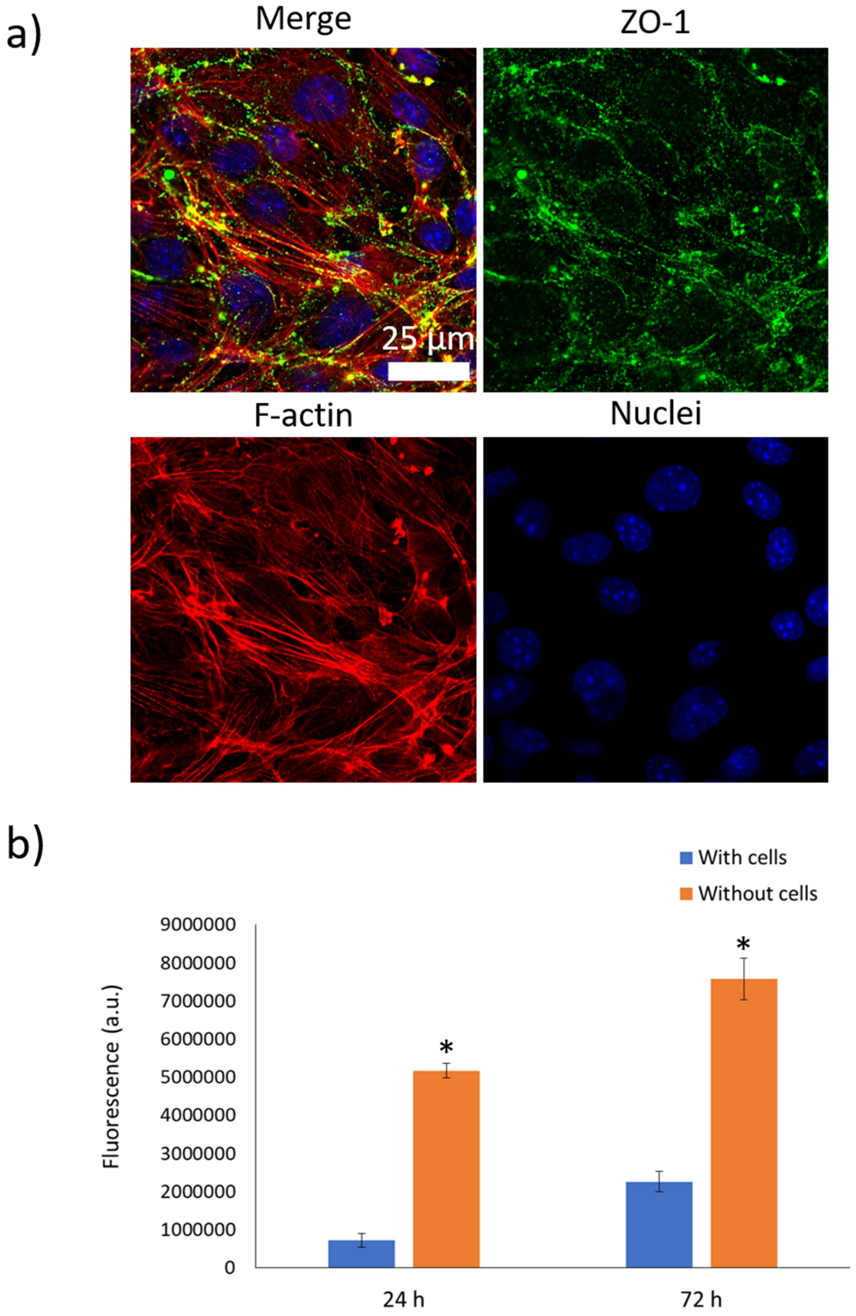

Figure S10. 2D confocal imaging (left) and 3D rendering (right) of a monolayer of bEnd.3 cells after the internalization of DiO-L-PDNPs (in red F-actin, in green DiO-L-PDNPs, in blue nuclei).

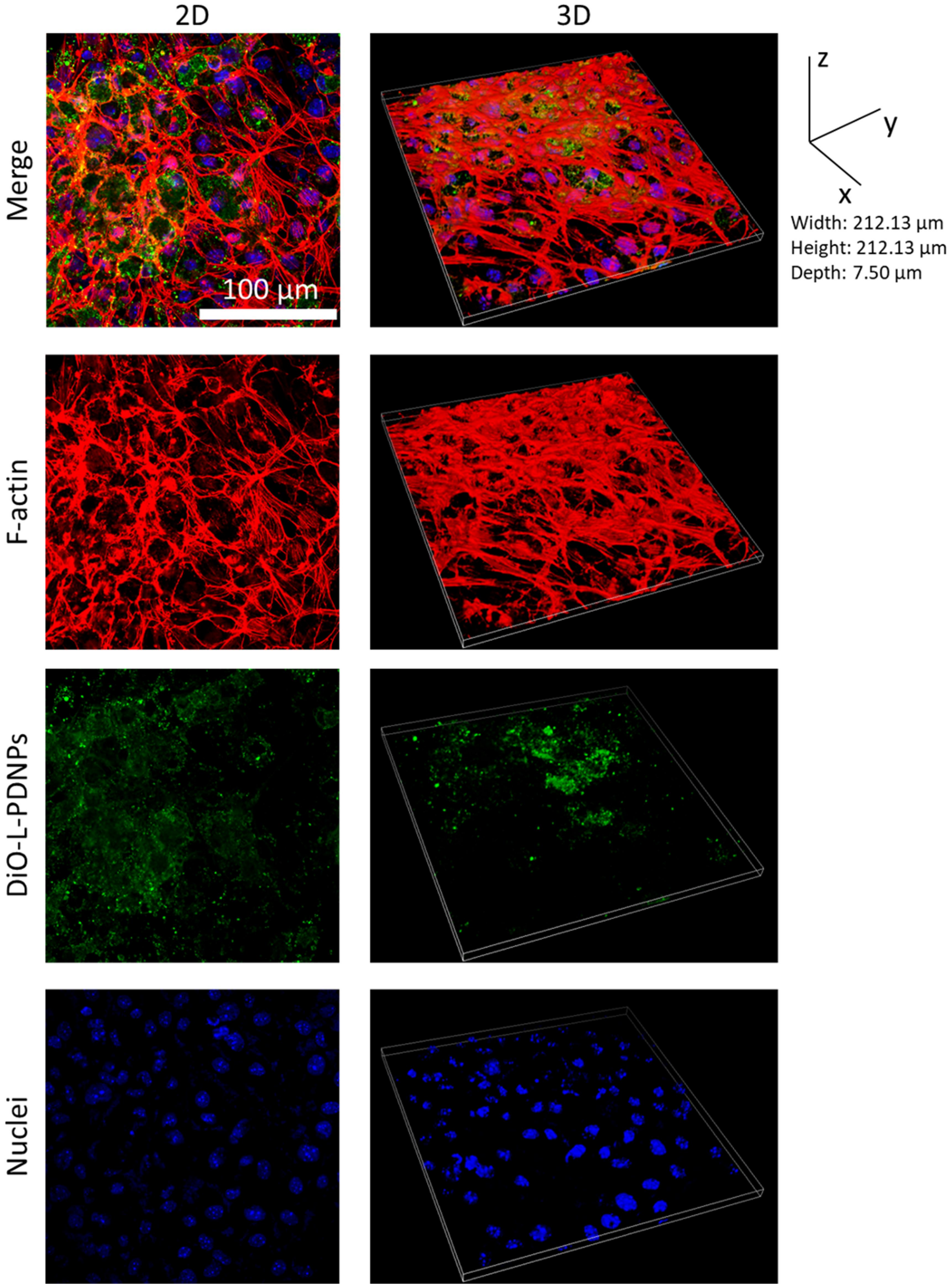

Figure S11. Representative flow cytometry data of differentiated SH-SY5Y treated with 100 µg/ml of L-PDNPs (and of controls), and subsequentially incubated for 4 h with different concentrations of TBH (0, 100, 500, 1000 and 5000 µM). Fluorescence levels derived from the double staining with annexin-V-FITC and propidium iodide are shown; the plots have been divided into four different quadrants basing on fluorescence thresholds (healthy cells in green, early apoptotic in yellow, late apoptotic in orange, and late apoptotic in red).

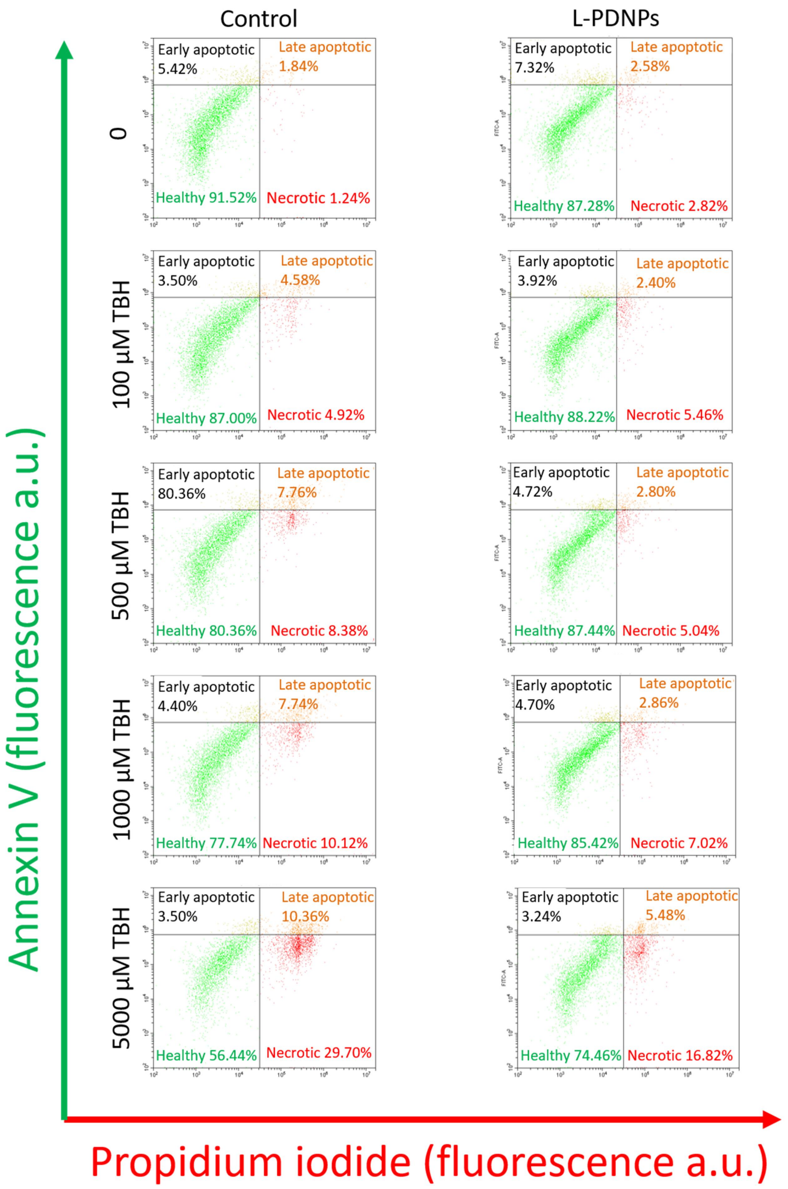

Figure S12. a) Absorbance profile of L-PDNPs at different concentrations, and b) respective values at 808 nm (wavelength of the laser used in this work). c) Heating profile of aqueous dispersions of L-PDNPs at different concentrations irradiated with NIR laser (808 nm, 532 mW power, 2.5 mm spot size).

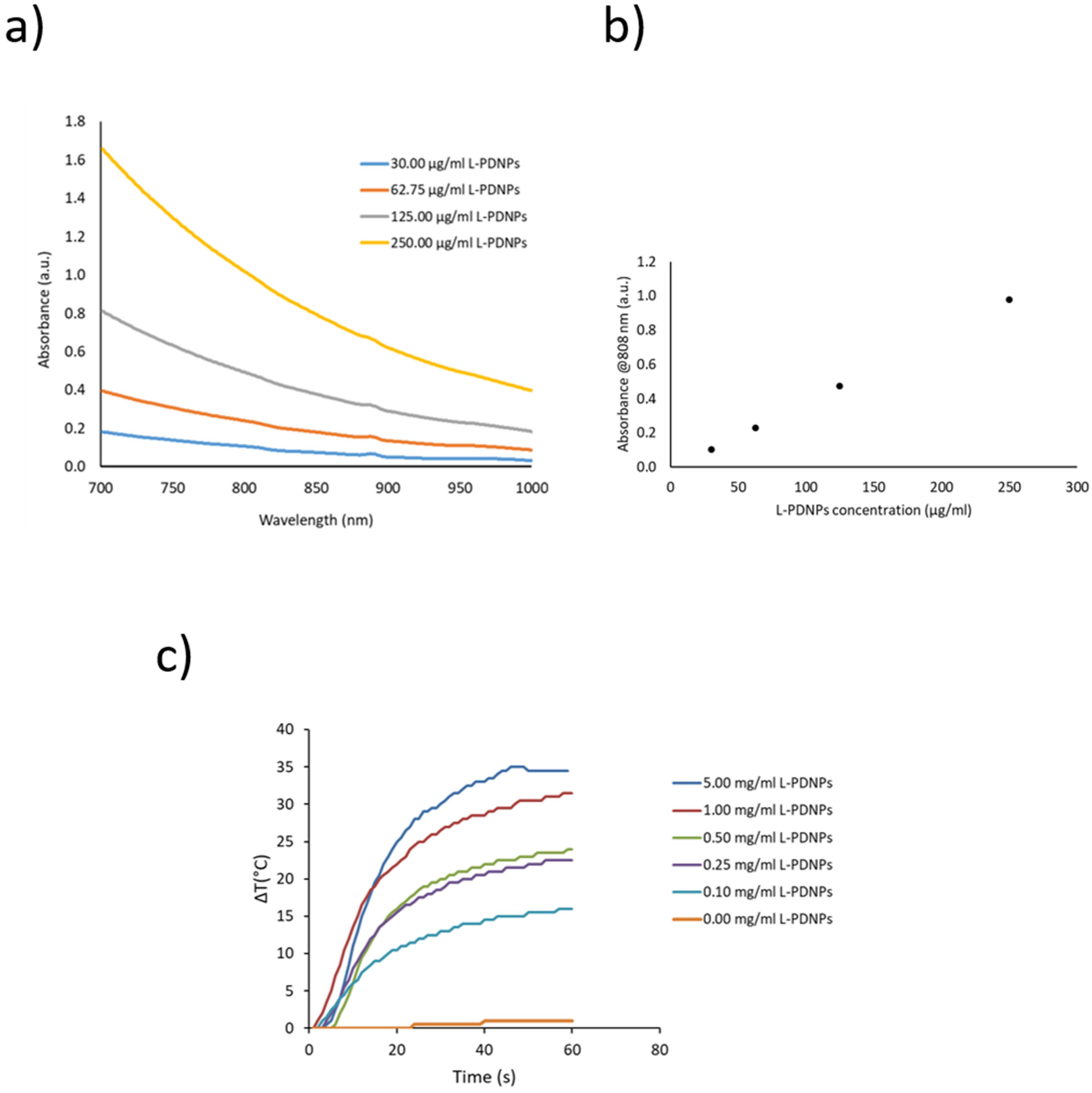

Figure S13. Analysis of ROS production in presence of NIR stimulation. a) Percentages of ROS-negative and ROS-positive cells in the different experimental classes (control, NIR, L-PDNPs and L-PDNPs + NIR). No statistically significant differences were observed (n = 3, p > 0.05 in all comparisons). b) Representative flow cytometry data of the performed analyses.

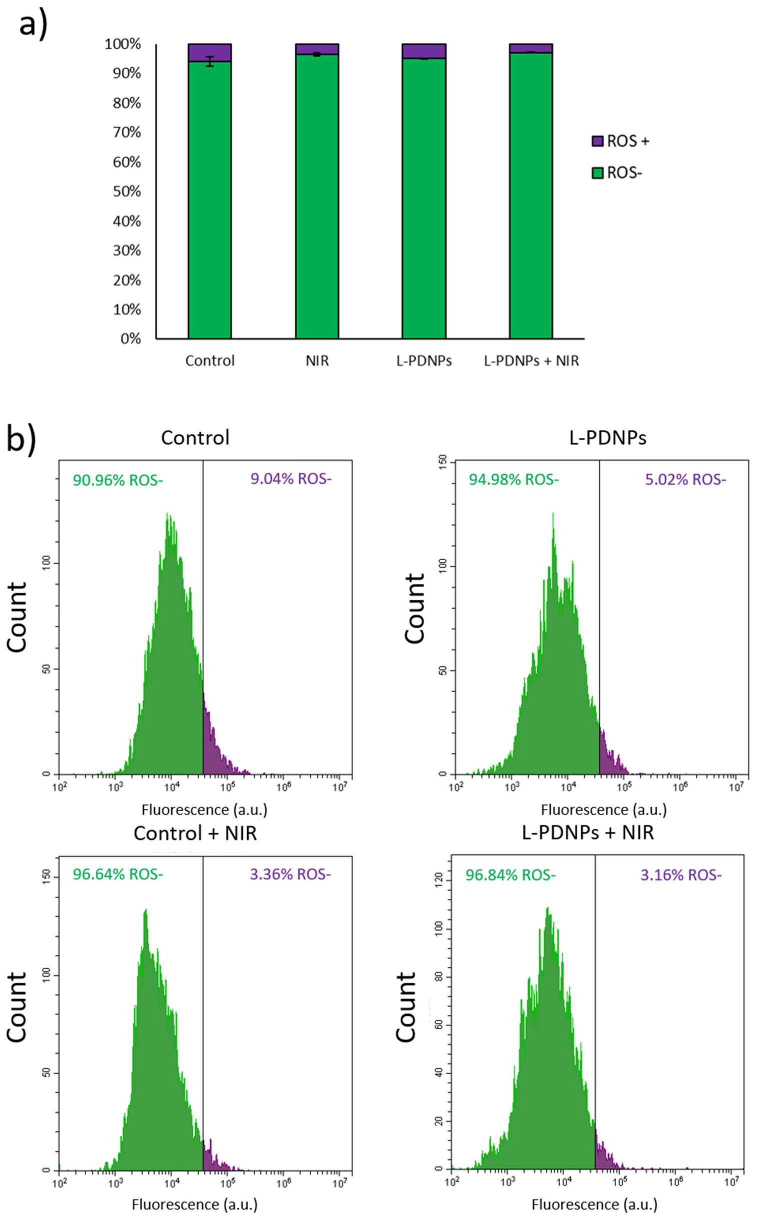

Figure S14. Model schematics. a) 3D cylindrical domain, with NIR irradiation aligned in such a way to allow for axisymmetric approximation. b) Derived 2D section highlighting relevant sizes. c) Derived 1D domain used to formulate the energy balance based on conduction (*q*) and thermal exchange (*te*) heat fluxes, and heat sources in the irradiated segment.

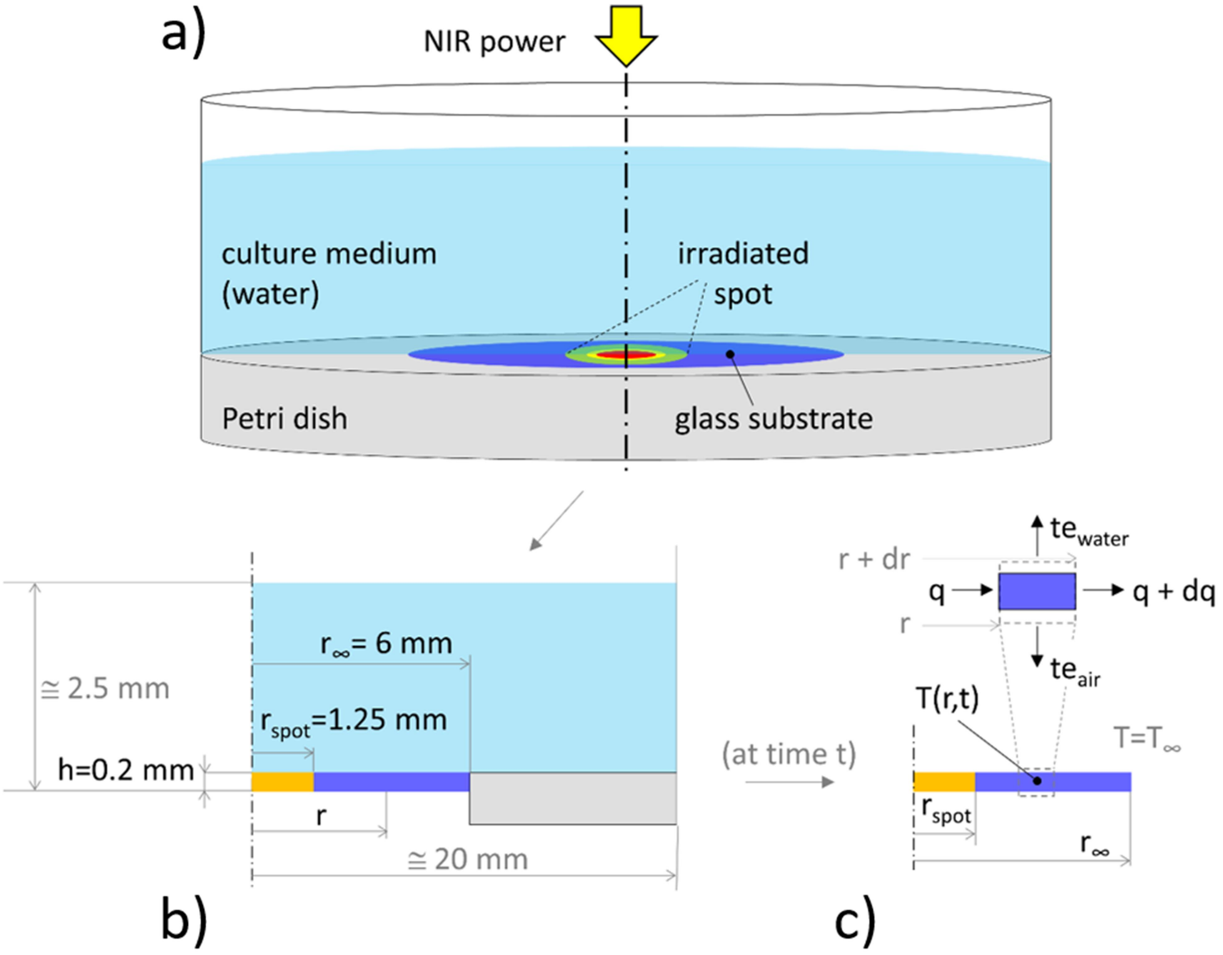

Figure S15. a) SEM image showing SH-SY5Y cells and the glass bottom of a Wilko Petri dish with associated L-PDNPs. b) Binarization of the image in a).

a)

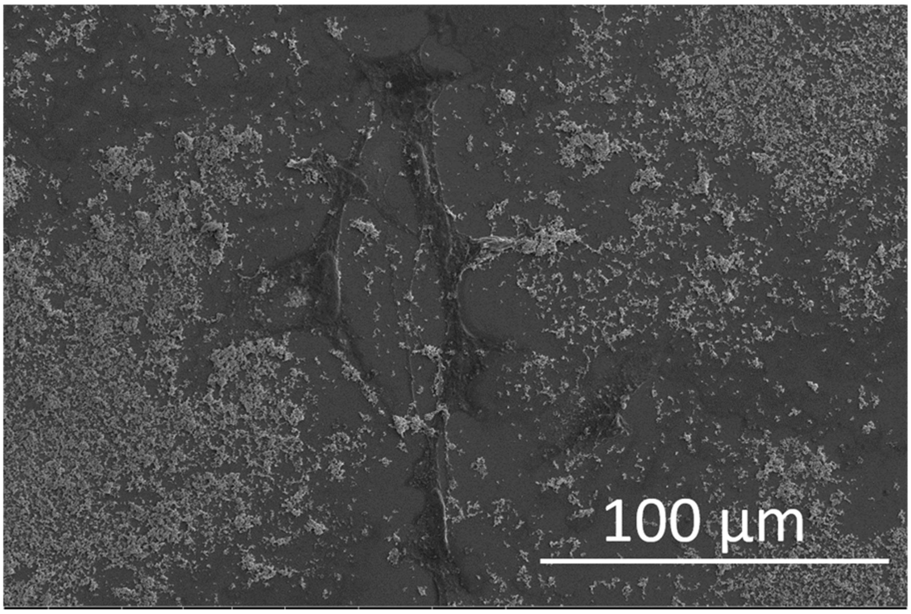

b)

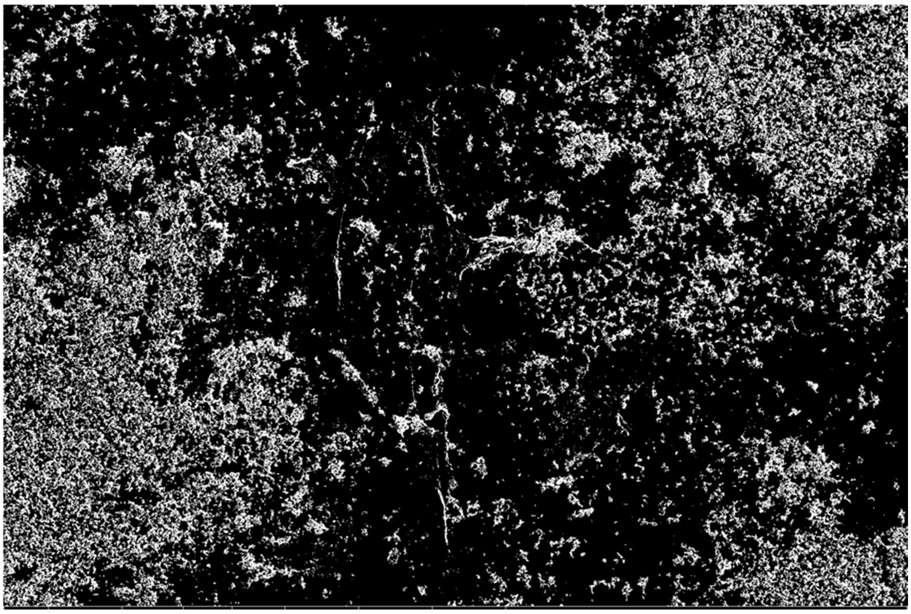

Supplement: Supplementary file 1 — am0c05497_si_001.pdf [file am0c05497_si_001.pdf]
